# A Molecular Docking Study of Human STEAP2 for the Discovery of New Potential Anti-Prostate Cancer Chemotherapeutic Candidates

**DOI:** 10.3389/fbinf.2022.869375

**Published:** 2022-05-24

**Authors:** Timothy Ongaba, Christian Ndekezi, Nana Nakiddu

**Affiliations:** ^1^ Department of Biomolecular Resources and Biolaboratory Sciences, College of Veterinary Medicine, Animal Resources and Biosecurity (CoVAB), Makerere University, Kampala, Uganda; ^2^ Uganda Virus Research Institute, Entebbe, Uganda; ^3^ Joint Clinical Research Centre, Kampala, Uganda; ^4^ College of Health Sciences (CHS), Makerere University, Kampala, Uganda

**Keywords:** prostate cancer, molecular docking, bioinformactics, computational drug discovery, STEAP2, AutoDock Vina

## Abstract

Prostate cancer is a rising health concern and accounts for 3.8% of all cancer deaths globally. Uganda has one of the highest incidence rates of the disease in Africa at 5.2% with the majority of diagnosed patients found to have advanced disease. This study aimed to use the STEAP2 protein (prostate cancer–specific biomarker) for the discovery of new targeted therapy. To determine the most likely compound that can bind to the STEAP2 protein, we docked the modeled STEAP2 3D structure against 2466 FDA (Food and Drug Administration)-approved drug candidates using AutoDock Vina. Protein basic local alignment search tool (BLASTp) search, multiple sequence alignment (MSA), and phylogenetics were further carried out to analyze the diversity of this marker and determine its conserved domains as suitable target regions. Six promising drug candidates (ligands) were identified. Triptorelin had the highest binding energy (−12.1 kcal/mol) followed by leuprolide (docking energy: −11.2 kcal/mol). All the top two drug candidates interacted with residues Ser-372 and Gly-369 in close proximity with the iron-binding domain (an important catalyst of metal reduction). The two drugs had earlier been approved for the treatment of advanced prostate cancer with an elusive mode of action. Through this study, further insight into figuring out their interaction with STEAP2 might be important during treatment.

## 1 Introduction

Cancer is a disease in which abnormal cells divide uncontrollably and progressively destroy body tissue. It is the second leading cause of death before 70 years in 91 of 172 countries according to the World Health Organization in 2015 and responsible for an estimated 9.6 million deaths in 2018 (Bray et al., 2018). One in 5 men and 1 in 6 women develop cancer in their lifetime while 1 in 8 men and 1 in 11 women die from the disease (Bray et al., 2018). As of 2018, the global cancer burden had risen to 18.1 million new cases (Bray et al., 2018). Worldwide, the total number of people alive with a 5-year cancer diagnosis, called the 5-year prevalence, was estimated to be 43.8 million (World Health Organisation, 2018). About 70%–80% of the avoidable cancer mortality occurs in low to middle income countries (LMICs) (Knaul et al., 2018). Global cancer incidence is on the rise and it is predicted that by 2050, 70% of the 24 million annual cancer diagnoses will be individuals residing in LMICs (Kingham et al., 2013).

Prostate cancer is the second most frequently diagnosed cancer in men globally and accounts for 3.8% of all deaths caused by cancer in men as of 2018 (Bray et al., 2018; Ferlay et al., 2018). In Uganda, for a 16-year period from 1991 to 2006, there was an increase in cancer risk particularly for breast and prostate cancer (4.5% annually) (Parkin et al., 2010). The incidence of prostate cancer in Uganda is among the highest recorded in Africa at 39.6 per 100,000 after age-standardizing (Parkin et al., 2010). A recent study on prostate cancer burden puts its annual incidence at 5.2% (Okuku et al., 2019). Prostate cancer can be classified into localized and metastatic disease depending on the absence or presence of spread respectively. Localized prostate cancer is classified as high risk based on clinical staging, prostate-specific antigen (PSA) levels, and/or the Gleason score (Caster et al., 2015). Patients with metastatic prostate cancer are at a higher risk of disease and death. The disease can be categorized as castration-sensitive (mCSPC) when surgical removal of the testicles halts cancer advancement due to decreased blood testosterone androgen or castration-resistant (mCRPC) when the cancer continues to progress even in the absence of testosterone androgen (Caster et al., 2015). The majority of mCSPC patients have a high risk of progressing to mCRPC when initial hormonal treatment fails due to resistance (Caster et al., 2015). Prostate cancer can be treated with multimodal therapy in a risk-adapted approach. Surgery, nanotechnology for controlled drug delivery, monoclonal antibody therapy, hormonal therapy, radiotherapy, and chemotherapy are some of the treatment methods (Held-Warmkessel, 2001; Nanus et al., 2003). Chemotherapy (especially when hormonal treatment fails), surgery, and radiotherapy are the most commonly used modalities of therapy.

Six transmembrane antigen of prostate 2 (STEAP2) also known as the six transmembrane protein of prostate 1 (STAMP1) is a member of the metalloreductase family important in the metal reduction of copper and iron. *In vitro* and *in vivo* studies show that STEAP2 plays a key role in prostate cancer progression (Whiteland and Claire, 2014). STEAP2 is located on the plasma membrane of prostate cells and Golgi complex. It increases prostate cancer progression, controls cell proliferation, differentiation, and decreases apoptosis (Grunewald et al., 2012). Its knockdown from prostate cancer cells has been shown to reduce their invasive potential, increased apoptosis, and reduced migration that are responsible for oncogenesis and disease progression (Wang et al., 2010a; Burnell et al., 2018). Immunohistochemical staining significantly demonstrates its expression at the cell–cell junctions of prostate cancer cells (Hubert et al., 1999). It is differentially expressed in normal and cancerous tissue making it a potential target for new therapeutic strategies for disease treatment (Gomes et al., 2012). STEAP2 is expressed more than 10 times in normal prostate than in other tissues such as the brain and liver and is exponentially expressed in malignant prostate cancer cells (Porkka et al., 2002a; Korkmaz et al., 2005). Their levels in these tissues are too low to have any functional significance (Porkka et al., 2002a). STEAP2 is highly expressed at all stages of prostate cancer and is androgen independent, a characteristic that is key in managing androgen-dependent and independent/advanced prostate cancer [(Gomes et al., 2012), (Hubert et al., 1999)]. Its unique and specific upregulation in cancerous prostate tissue at all stages is likely to make it an ideal therapeutic drug target.

The exact STEAP2 mechanisms and pathways involved in the development and progression of the disease, however, remain poorly understood in addition to other STEAP proteins and warrant further investigation. The epithelial–mesenchymal transition (EMT) process and phoshpatidyinositol-3-kinase protein kinase B mammalian target of rapamycin (PI3K/Akt/mTOR) signaling pathways seem to be implicated (Yang et al., 2019; Wu et al., 2020).

Wang et al. (2010b), though inconclusively, found that STEAP2 may influence the progression of prostate cancer by activating the extracellular signal–regulated kinase signaling pathway, the mitogen-activated protein kinase (MAPK) pathway, cell cycle progression, and inhibition of apoptosis pathways. The exact regulatory mechanism of synergism or antagonism of the pathways in prostate cancer remains not clearly understood (Guo et al., 2020)

Although there has been great improvement in therapeutic options for prostate cancer over the last decade, the drugs currently used are still limited and not 100% effective making the cure elusive (Caster et al., 2015). This is further compounded by undesirable side-effects from some of the treatments. Altogether this creates a need for the discovery of novel, safe, and efficacious chemotherapeutic agents with minimal or no side effects. Rational drug design if informed by drugs that target prostate cancer-specific protein biomarkers, can help improve drug specificity, efficacy, and reduce undesirable side effects. STEAP2 metalloreductases might be one such prostate-specific protein biomarker and drug target that may be studied in. This study aimed at using the STEAP2 prostate cancer biomarker as a target using *in silico* methods and promising results were achieved. STEAP2 targeting may be studied in the future in the context of developing anti-prostate cancer therapies.

## 2 Materials and Methods

### 2.1 Study Design

**FIGURE 1:**
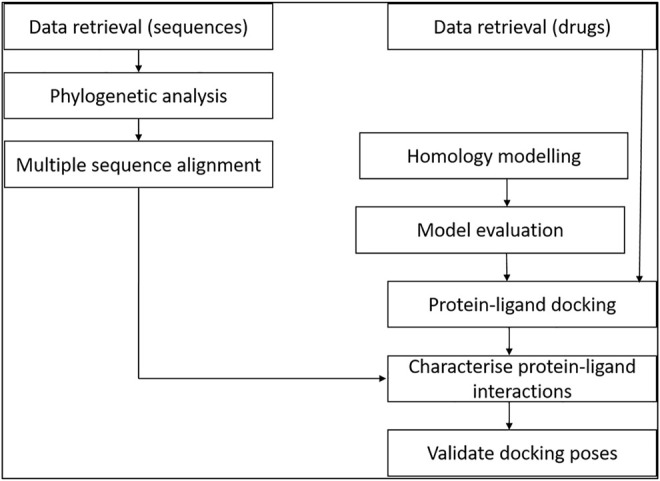
Study design of the methodology. The relationship of data from multiple sequence alignment of related species to human STEAP2 gave insight on key protein residues important in function of the protein to look out for in ligand interaction with the protein during protein–ligand docking.

The study involved retrieval of STEAP2-related protein sequences from the protein data bank from a protein BLAST search. The selected sequences were used for multiple sequence alignment (MSA) and phylogenetics to determine the most related species to the human STEAP2 protein. For molecular docking, a set of FDA-approved drugs were retrieved from the drug bank and used as ligands for protein–ligand docking. The protein (STEAP2) 3-D structure was first modeled and evaluated prior to the docking exercise. The protein–ligand interactions were characterized using ligplots (Figure 1).

### 2.2 STEAP2 Relative Tissue Expression

To determine the expression of STEAP2 protein in different tissues, an expression analysis was conducted using the Human Protein Atlas (https://www.proteinatlas.org/ENSG00000157214-STEAP2) (Uhlén et al., 2015). The search term “human STEAP2” was used to find the expression of the protein in different tissues within the body.

### 2.3 Data Retrieval

The amino acid sequence of the query protein *H. sapiens* STEAP2 (Uniprot accession number: Q8NFT2) was obtained in a FASTA format from UniProt (Bateman et al., 2017). This sequence was used in the protein BLAST search for homologous sequences in the PDB.

### 2.4 STEAP2 Multiple Sequence Alignment and Phylogenetic Analysis

The MSA was performed in JalView using 30 selected FASTA files from a non-redundant protein BLAST (BLASTp) search of the reference proteins in the PDB. In the BLASTp search, the algorithm parameter of maximum target sequences to return was adjusted to 5,000 and the rest of the parameters were left as default. A total of 30 sequences of the 5,000 returned were selected for MSA based on the percentage similarity in bins of 0–40, 41–60, 61–80, and 81%–100%. A number of sequences were selected from each range (Table 1).

**TABLE 1:** Sequences selected for phylogenetic analysis of STEAP2 by MSA.

Similarity range (%)	Sequences selected
0–40	8
41–60	8
61–80	7
81–100	7

From the table, the 0–40 and 41–60 similarity ranges took an extra hit to ensure that more distant hits from the query sequence were considered for confidence purposes.

To generate an evolution tree/dendrogram, a new alignment of the amino acid sequences from FASTA files of STEAP proteins of 30 species from the BLASTp search and subsequent 15 (only STEAP2 containing) was created *via* the align tab in MEGAX (Kumar et al., 2018). The sequences were copied, pasted, and alignment was performed on all using Muscle with all alignment parameters left as default. The output alignment file was used as an input for phylogenetic analysis. The maximum likelihood tree was used. In the analysis preferences, the bootstrap method was used and the number of replications were adjusted to 1,000. The (Lo and Gascuel)+discrete gamma distribution of rates across sites (G)+evolutionarily invariable sites (I) model was used and the rest of the parameter left as default. A dendrogram showing a phylogenetic relationship of STEAP2 was generated.

#### 2.4.1 Homology Modeling

*H. sapiens* STEAP2 (Uniprot accession number: Q8NFT2) FASTA sequence was used as a query sequence in the different modeling engines that were best-ranked using the Continuous Automated Model Evaluation (CAMEO) website and one other from the critical assessment of protein structure prediction (CASP) website (I-TASSER) to search for similar sequences for homology modeling (Haas et al., 2019; Kryshtafovych et al., 2019). The similarity of retuned results from the BLAST was shown in terms of coverage of the query sequence, the percentage identity, and the E-value. Also, the organism that was the source of the sequence and the PDB identification of each result was shown. The best hit’s resolution and method of structural determination were obtained from the protein data bank (Bernstein et al., 1977). The best sequence in all but those of RaptorX and I-TASSER were manually chosen for homology modeling. I-TASSER automatically chose the best nine sequences and made the homology models of STEAP2 using each, and the best-ranked result (homology model 1) was chosen as the final homology model. RaptorX automatically chose its best template and showed its homology model as a result at the end of the process. The top templates by the rest of the engines were used to model STEAP2 using Modeler and ProMod3 (Webb and Sali, 2017; Waterhouse et al., 2018). The homology models were downloaded as PDB files and visualized using PyMol and Chimera (Pettersen et al., 2004; Schrödinger and DeLano, 2020)

#### 2.4.2 Model Evaluation of Homology Models

The homology models from the different modeling engines were evaluated using different structural model evaluation tools: ProSa, QMEAN, Rampage, DOPE scores, and RMSD. Ramachandran plot server (URL: https://zlab.umassmed.edu/bu/rama/index.pl). Using PyMol, the stoichiometry of the homology model was determined by chain coloration and critically looking at structures to identify any non-conformity such as long loops or knots (Schrödinger and DeLano, 2020). RMSD with the 6HCY (human STEAP4) template was calculated using the PyMol alignment tool to align the template with the homology models to determine average structural deviations of the homology model from that of the used template. The DOPE score of each homology model was calculated using Modeller. Z-DOPE scores were calculated for individual homology models using Modeller (Shen and Sali, 2006; Webb and Sali, 2017)

### 2.5 Molecular Docking

With the best model selected from 6 STEAP2 homology models after evaluation, the homology model by SWISS-MODEL was used for the flexible ligand, rigid receptor protein–ligand docking using a group of approved drug compounds downloaded from the drug bank. These compounds were downloaded as a SANCDB file of 2466.

### 2.6 Protein–Ligand Docking Using AutoDock Vina

The ligand and receptor molecules were prepared using prepare_ligand4.py and prepare_receptor4.py, respectively, by command line on Linux terminal. The two python scripts add polar hydrogens (Q) and define atom types (T) in the ligand and receptor files (in a PDB format), respectively, to generate their PDBQT files. The search box in the configuration file was set to dimensions x, y, and z (105 by 105 by 105 Å, with a center at: x = 122.224 Å, y = 118.600 Å, and z = 102.658 Å). These were determined using MGL tool graphical user interface with cross-sectional measurements along each axis. The median point of the line between the two furthest points of the molecule on that axis guided toward the center point to center the search box so as to cover the entire protein in all 3 dimensions.

The results of molecular docking were characterized by 3D proximity to a central residue termed as “Euclidian distance”— which is the length of a line between two points in geometrical space which can be 2 or 3D and binding energy of the ligand to the receptor molecule. All results were screened and a binding energy threshold of less than or equal to −7 and Euclidian distance of 50 Å were set. The complexes that met the selection criteria were then considered for further analysis.

The entire protein was covered by the search box during molecular docking, a method also known as blind docking to mitigate bias to any region of the receptor.

### 2.7 Characterizing Protein–Ligand Interactions

To determine the nature of the bonds formed between STEAP2 and the different ligands, Ligplot which is a program used to generate 2D representations of the nature of bonds formed between protein receptor residues and ligand atoms was used for this task (Wallace et al., 1995). The program showed the hydrogen bond interactions in green dotted lines with their respective lengths in angstroms and the different interacting protein residues in an arc shape with spokes radiating toward the atoms they are in contact with. The interactions not only accounted for hydrogen bonds but all hydrophobic interactions between the two molecules (Wallace et al., 1995).

### 2.8 Docking Validation Using Independent Docking Engines

The order of binding energy was validated using PatchDock server and AutoDock4. (Huey and Morris, 2005; Schneidman-Duhovny et al., 2005). The receptor PDB file and ligand pdbqt files were uploaded on a PactchDock server. In AutoDock4, the protocol in the tutorial by Huey *et al.* was followed (Huey and Morris, 2005). The binding site of STEAP2 was validated using Protein Binding Site (ProBiS) and PrankWeb webservers (Konc and Janežič, 2010a; Jendele et al., 2019). Chain A of the homo-3-mer without its HETATOMs and water molecules was the input file and was uploaded in a PDB format. All other parameters were left as default and the job was submitted for analysis at both submission webpages. The druggability of the protein was assessed using CAVIAR in the Linux terminal to identify potential cavities for ligand binding based on scores and size and ligandability among other criteria (Marchand et al., 2021). The protein chain A PDB file was used as the input file.

## 3 Results

### 3.1 STEAP2 Relative Tissue Expression

**FIGURE 2:**
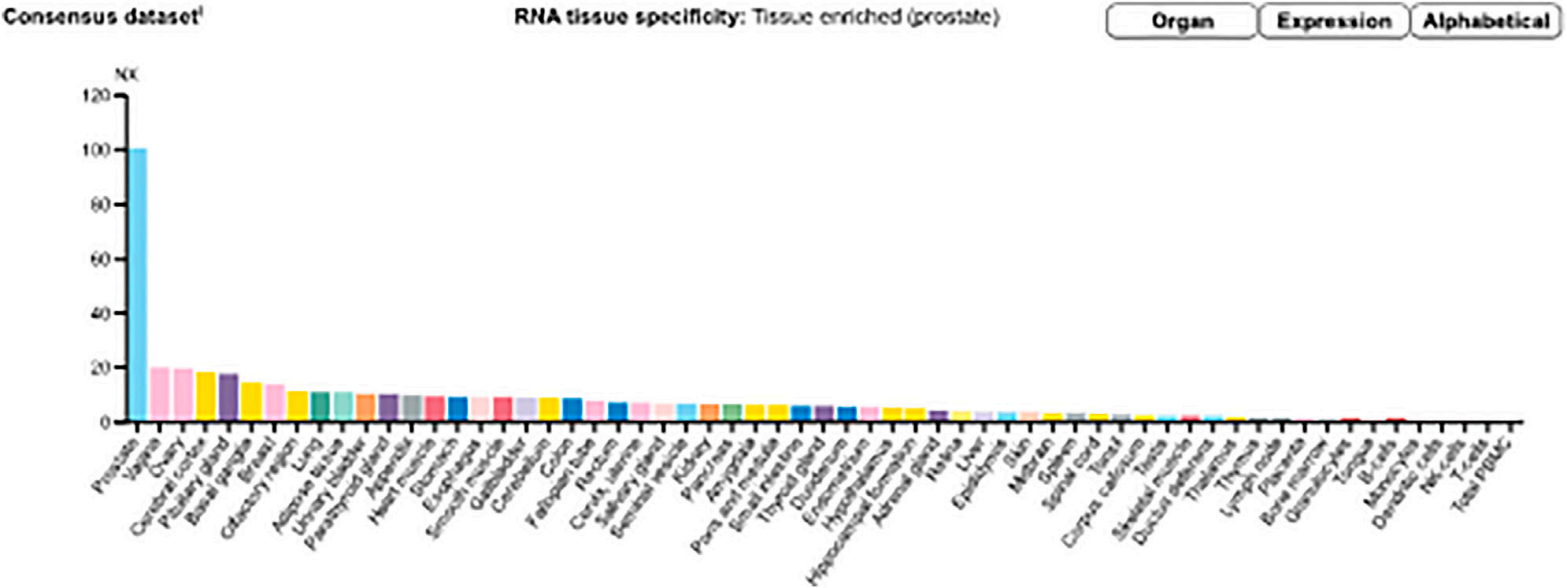
Consensus normalized expression (NX) levels for 55 tissue types and six blood cell types, created by combining the data from the three transcriptomics datasets (HPA, GTEx, and FANTOM5) using the internal normalization pipeline. (Source: https://www.proteinatlas.org/ENSG00000157214-STEAP2/tissue). The graph maintains the initial observation that prostate tissue contains significantly high amounts of STEAP2 that any other body tissue of the 55 considered.

Messenger ribonucleic acid (mRNA) expression analysis of the FANTOM5 dataset showed that STEAP2 was most abundantly expressed in prostate tissue followed by ovarian and vaginal tissue (Supplementary Figure S2). Prostate tissue had the most STEAP2 in mean protein coding transcripts per million as compared to all other listed body tissues using the HPA dataset (Supplementary Figure S3). The analysis of STEAP2 mRNA transcripts from different tissue in mean protein coding transcripts per million in comparison to 33 other listed tissues using the GTex dataset showed prostate tissue to have the highest quantity of STEAP2 (Supplementary Figure S4). The three datasets were combined by a normalized expression level and showed the same trend (Figure 2).

### 3.2 STEAP2 Phylogenetic Analysis

**FIGURE 3:**
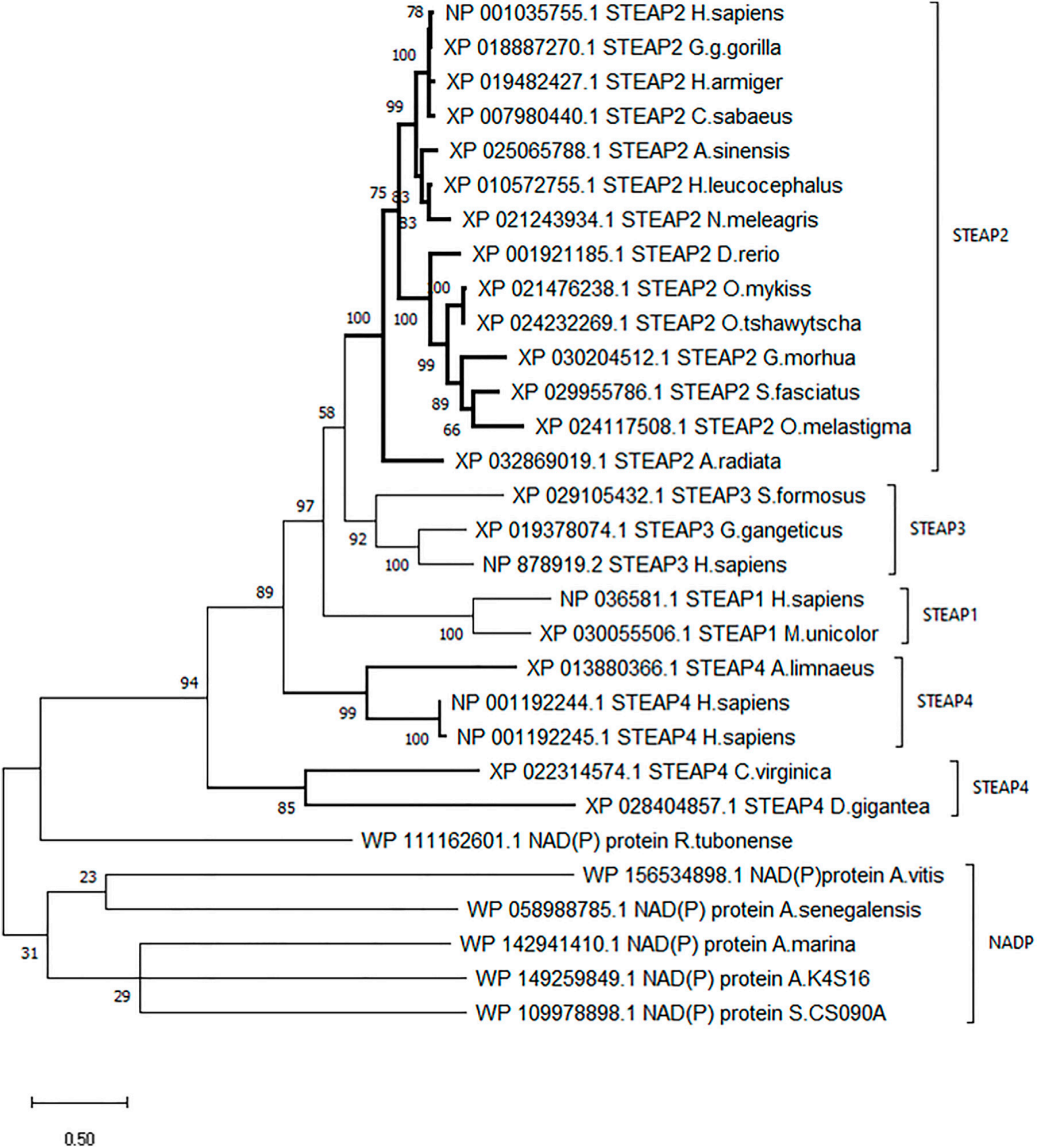
Phylogenetic tree of *H. sapiens* STEAP2 shows that it is most related to *G. gorilla* since it is redrawn 78% of 1,000 times by the bootstrap method. This, therefore, indicates that these species’ have the most conserved regions.

A dendrogram generated using Molecular Evolution Genetic Analysis (MEGA) showed that STEAP2 was conserved mostly in species under phylum Chordata (Figure 3). The values at each node indicate the percentage times of 1,000 that particular node was redrawn as a result of genetic relationship of its taxons.

The STEAP2 containing species clustered alone in one clade and also happen to be under phylum Chordata, *H. sapiens* falls together with its most similar species *G. gorilla* (Figure 3). The rest of the clades contain other STEAP proteins and one NADP from other classes such as Pisces.

**FIGURE 4:**
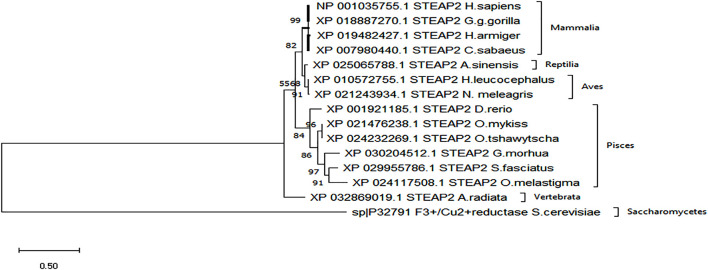
From the phylogenetic tree, which is a result of 1,000 bootstraps, it is shown that the STEAP2 protein from *G. gorilla* (mammal) is most related to human STEAP2 from 99% of the 1,000 redrawn trees and the least related is *A. radiata*. The first four listed species from top belong to class Mammalia, followed by an alligator (Reptilia), birds (Aves) fish (Pisces), and a turtle (Vertebrata) all under phylum Chordata.

A secondary phylogenetic tree of only STEAP2s further demonstrated that the mammalian species baring STEAP2 clustered together, followed by those of Reptilia, Pisces, and Vertebrata species, *A. radiata* with the tree having been rooted to a yeast species *S. cerevisiae* (Figure 4).

### 3.4 Analysis of Highly Conserved Residues From the Multiple Sequence Alignment That Might be Responsible for the Phylogenetic Relationships of the Different Species

**FIGURE 5:**
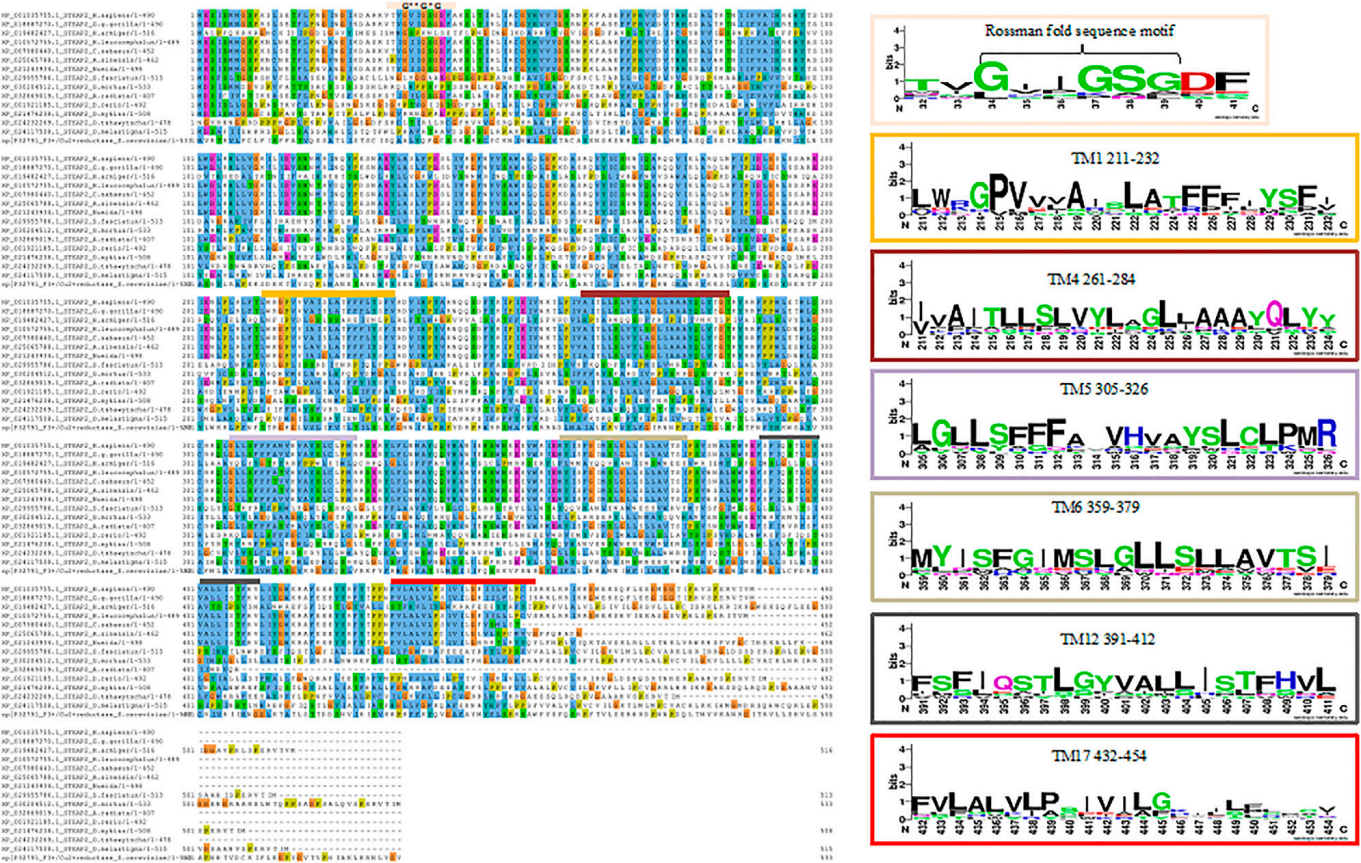
MSA of STEAP2 proteins from 14 species from 4 different classes of chordates. The most conserved residues are associated with functional roles of the protein. From top, the Rossman fold, an alpha beta alpha secondary structure with sequence GxGxxG is closely associated with the dinucleotide phosphate group of NADP followed by the first trans-membrane domain (TM1) (Hanukoglu, 2015). The second, third, fourth, and fifth TM domains (TM4, 5, 6, and 12, respectively) in close contact with the iron binding TM domain and finally the sixth TM domain.

The MSA image depicts the conserved residues across 15 selected sequences of STEAP2 baring species and *S. cerevisiae* from the original 30 from the protein BLAST search. The local regions that are highly conserved are shown with respective weblogos including the Rossman fold (amino acids residues 33–38) closely associated with the NADP domain and six other trans-membrane (TM) domains (TM1 with residues 211–232, TM4 with residues 261–284, Tm5 with residues 305–326, TM6 with residues 359–379, TM12 with residues 391–412, and TM17 with residues 432–454) associated with iron binding (Figure 5). An MSA image of all the 30 sequences is shown in Supplementary Figure.S1

### 3.5 Homology Modeling

Template 6HCY/6HDY (human STEAP4) was the most common top hit for five of the six modeling engines with a better coverage than 2VNS identified using PRIMO (Table 2).

**TABLE 2:** Top homology modeling templates from selected model engines and respective parameter of the hits.

Engine	Sequence identity (%)	Coverage	Organism	PDB ID	Stoichiometry	Resolution (Å)	Structural determination
HHpred	46.00	18–171	*H. sapiens*	6HCY_A	Homo-3-mer	3.1	EM
SWISS-MODEL	47.61	30–466	*H. sapiens*	6HCY.1.A	Homo-3-mer	3.1	EM
Primo	53.00	33–215	*H. sapiens*	2VNS	Homo-2-mer	2.0	X-ray Diffraction
Raptorx	—	31–466	*H. sapiens*	6HD1C	Homo-3-mer	3.8	EM
Robetta	47.83	1–490	*H. sapiens*	6HCYA	Homo-3-mer	3.1	EM
I-Tasser	47.90	8–90	*H. sapiens*	6HCYC	Homo-3-mer	3.1	EM

**FIGURE 6:**
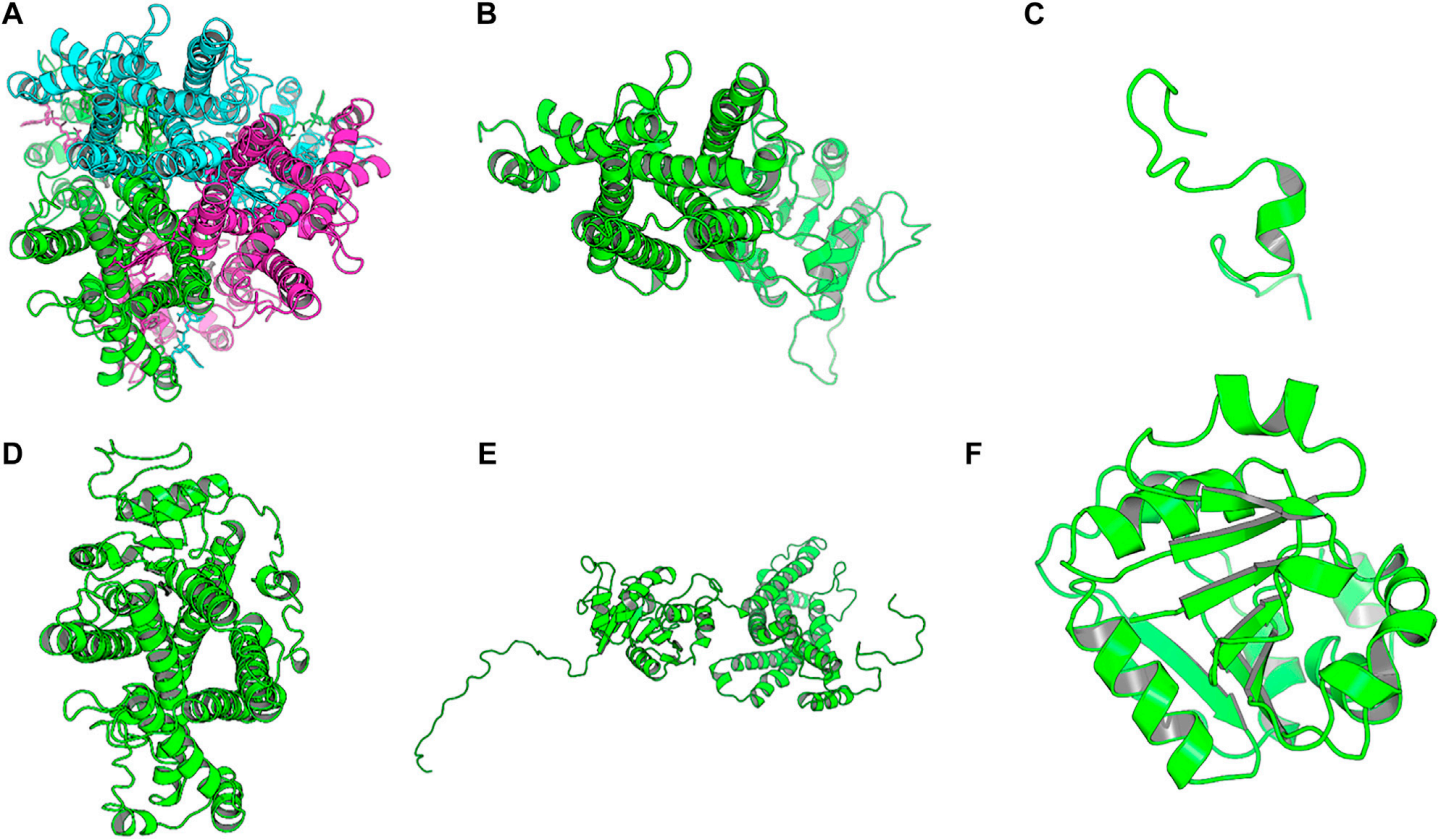
Images of the homology models were shown in cartoon format and colored by chain. Image A shows the homology model by SWISS-MODEL, **(B)** top homology model by I-Tasser, **(C)** homology model by HHPRED, **(D)** homology model by RaptorX, **(E)** homology model by Robetta and. **(F)** homology model by Primo. Only the homology model by SWISS-MODEL was a homo trimer, the rest were single chain models and the used template; human STEAP4 is a homotrimer.

The homology model from SWISS-MODEL was a homo-3-mer. Homology models from the different engines (images generated in cartoon format using PyMol) (Figure 6).

The homology model from SWISS-MODEL (Figure 6A) was a homo-3-mer made up of 437 amino acids per chain. The template used was 6HCY and the homology model had the least RMSD of 0.086 when superimposed with the 6HCY template (Table 3). Additionally, on average it had the best score by the used evaluation tools. For example, the Modeller normalized DOPE score of−5.95, the Ramachandran plot having 94.4 of its residues lying within the favored region a−3.06, and−6.33 Z-score from QMEAN and ProSA, respectively. Altogether, these results show that the SWISS-MODEL homology model was most similar model to the used template (Table 3). The comparative results of the SWISS-MODEL homology model and ab initio structural folding using AlphaFold2 are shown in Supplementary Figure S5 and a respective table. The results suggest that the homology model was a better predicted structure of human STEAP2 than the structure from AlphaFold2 that was derived by ab initio protein folding, a method better suited for query sequences with no or very low sequence identity to structurally solved sequence structures.

**TABLE 3:** Structural evaluation of homology models by different validation tools.

Modeling engine	Model stoichiometry	ProSA (Z-Score)	QMEAN (Z-Score)	Favored allowed outlier	Rampage	Allowed	Modeler (normalized Dope Z Score)	Aa modeled	RMSD with 6HCY
HHpred	Monomer	−6.33	−3.03	96.7	2.2	1.1	0.454	448	1.445
SWISS-MODEL	Homo-3-Mer	−6.33	−3.06	94.4	4.8	0.2	−0.595	437	0.086
I-Tasser	Monomer	−6.95	−6.36	87.5	9.8	2.7	−0.238	490	0.339
Raptorx	Monomer	−6.06	−2.91	96.7	2.7	0.6	0.867	487	0.367
Robetta	Monomer	−0.59	−0.19	88.0	12.0	0.0	0.285	28	3.789

The rest of the results are summarized in Table 3.

From Table 3, SWISS-MODEL on average had the best homology model after validation by the listed validation tools.

**FIGURE 7:**
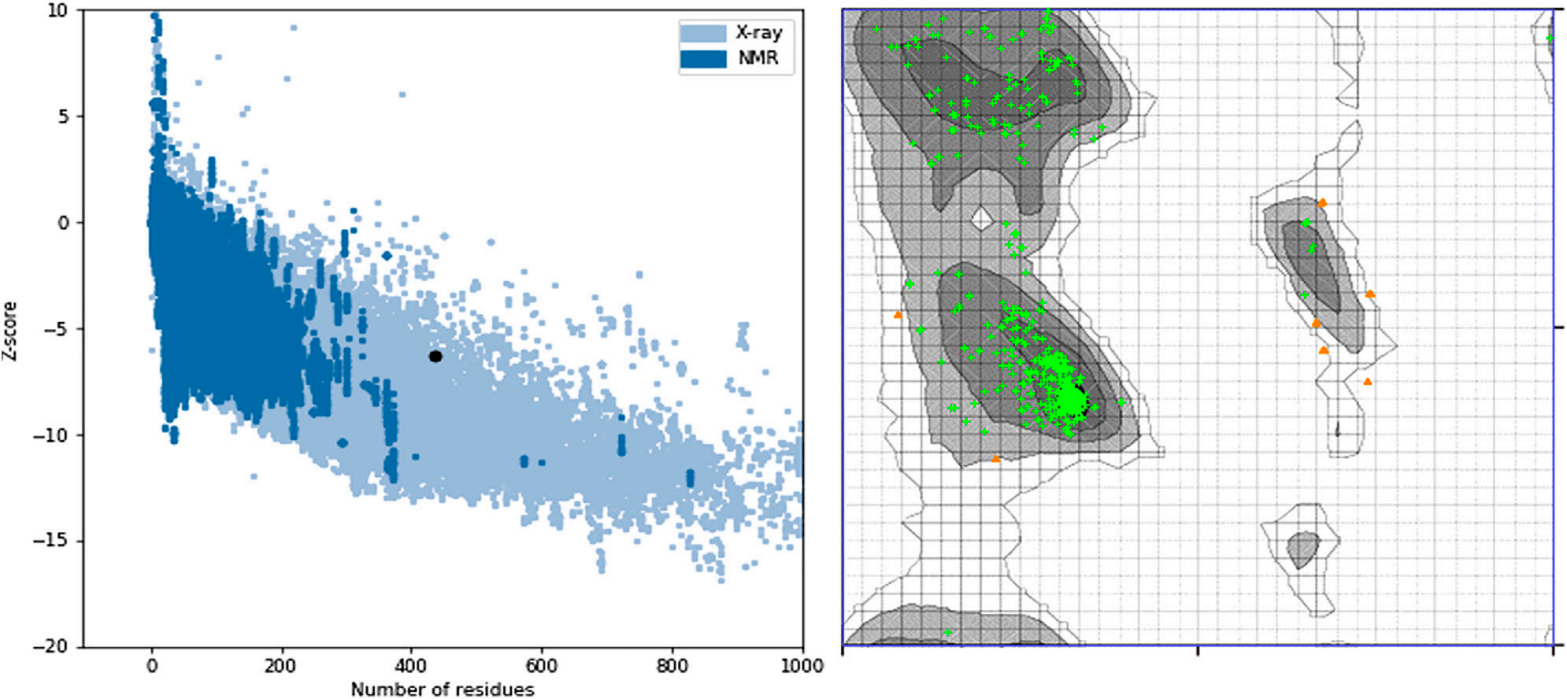
ProSA z-score and Ramachandran plot of the SWISS-MODEL homology model. The z-score of −6.33 suggests that the homology model is properly folded and 96.7% of the protein residues being under the favored region shows that almost all the protein residues are properly sterically placed in 3D space.

Figure 7 showed the ProSA evaluation of SWISS-MODEL which was the best homology model together with its Ramachandran plot on the right. It had a z-score of−6.33.

### 3.6 Molecular Docking

**FIGURE 8:**
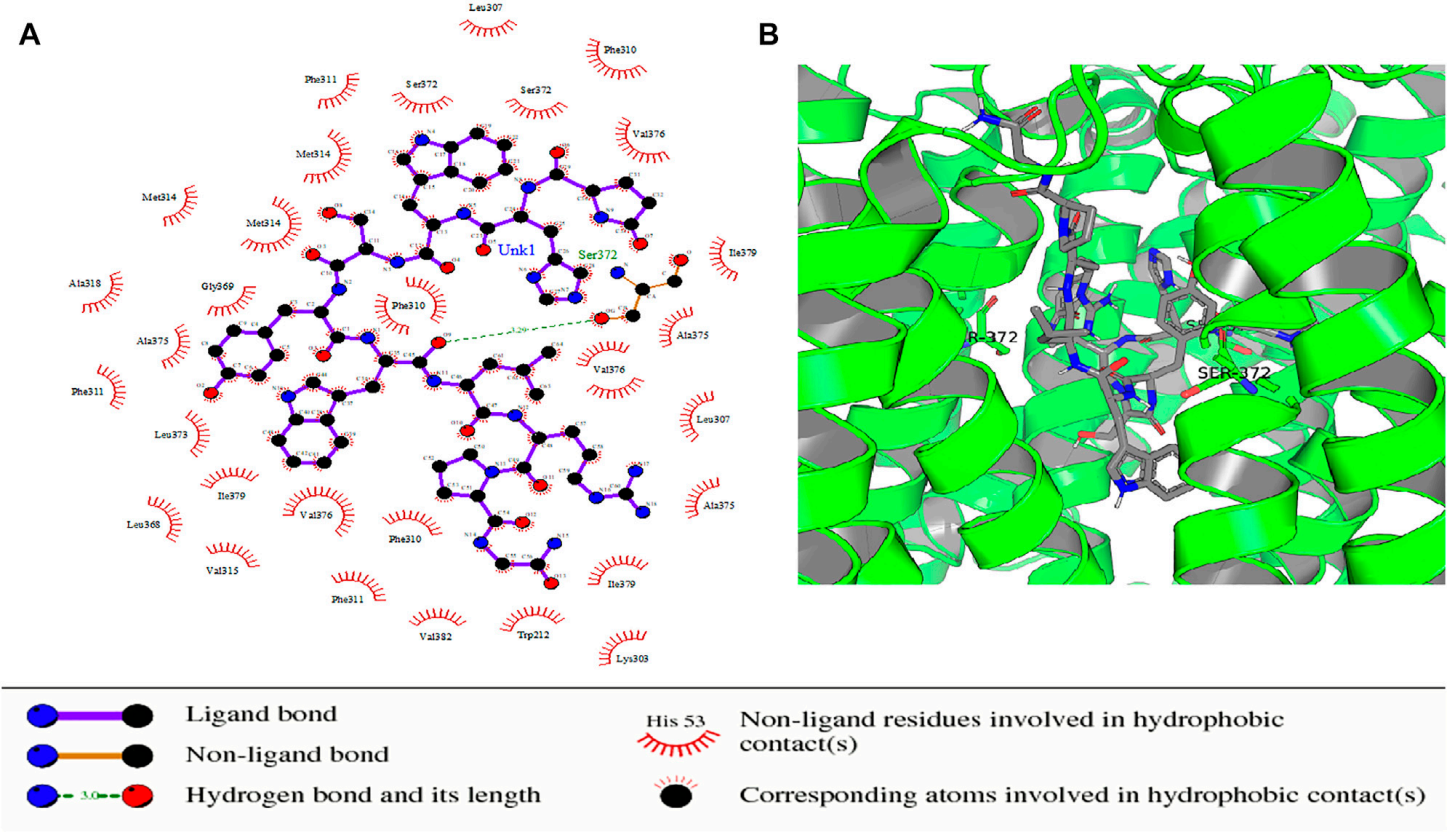
Protein–ligand complex of STEAP2 and Drugbank1543 shows residue Ser-372 from the 6th TM domain according to the MSA of STEAP2s and this forms a hydrogen bond with an atom of the ligand (Drugbank 1543). The rest of the interacting receptor residues are shown as crescents and these form other bonds with the receptors. These interacting residues are many compared to other complexes and contribute a significant binding energy of the complex.

**FIGURE 9:**
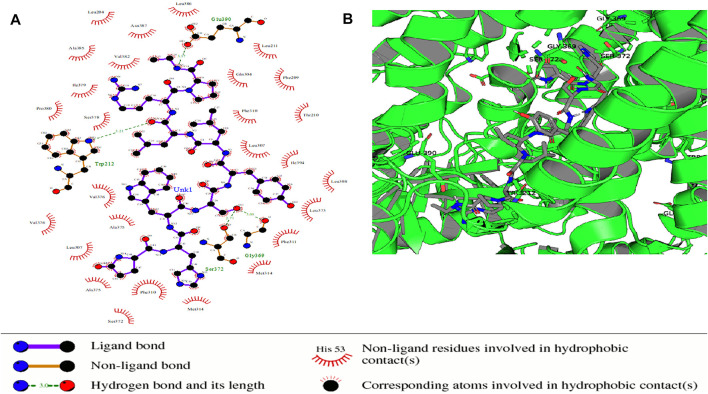
Protein–ligand complex of STEAP2 and Drugbank 2 shows residues Glu-390, Gly-369, Ser-372, and Gly-369 within the 6th and 12th TM domain form hydrogen bonds with ligand Drugbank 2.

**FIGURE 10:**
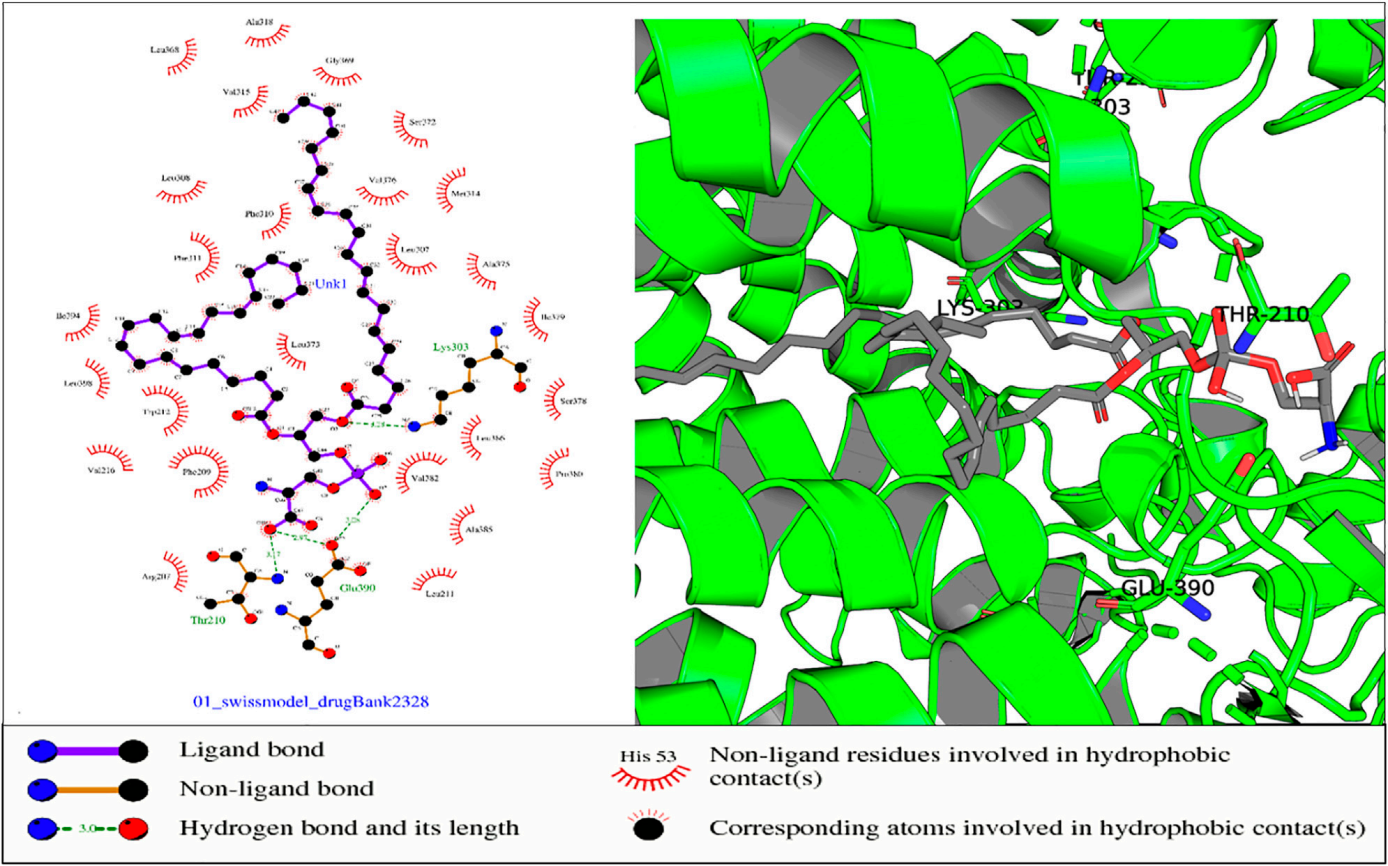
In the aforementioned complex, receptor residues Thr-210 (NADP domain), Glu-390, and Lys-303 (TM5) form hydrogen bonds with ligand Drugbank 2328.

Some of the receptor residues interacting with the drug ligands from the ligplots were found to be within highly conserved motifs of the STEAP2 protein with some forming hydrogen bonds in addition to other hydrophobic interactions. The 2D interactions are shown in panel A and the respective 3D in panel B of the images. The images of the top 3 complexes, Figures 8–10, are shown while the remaining three are attached as Supplementary Figures S6–8.

In the protein–ligand complex with Drugbank 1543 (triptorelin), Ser-372 with the hydrogen bond lies in the 6th TM domain which is part of the iron-binding domain and this is shown to be highly conserved from the MSA of only STEAP2 containing species (Figure 5). It together with many other shown protein amino acids binds with triptorelin a drug approved for the treatment of advanced prostate cancer (Figure 8).

In the protein–ligand complex with Drugbank 2 (leuprolide), the interacting protein amino acids such as Ser-372 with leuprolide lie within the TM domains that are highly conserved as shown in the MSA. These are associated with the iron-binding domain and bind to the ligand leuprolide which is also approved for the treatment of advanced prostate cancer (Figure 9).

In the protein–ligand complex with Drugbank 2328 (1,2 icosapentoyl-sn-glycero-3-phosphoserine), some of the listed residues such as Thr-210 are conserved under the NADP domain, the 5th TM domain having residues Glu-390 and Lys-303. The residues together with others form bonds with ligand Drugbank 2328 which is 1,2 icosapentoyl-sn-glycero-3-phosphoserine, an approved experimental drug for Corona virus-19 disease treatment (Figure 10).

Residues from Supplementary Figures S6 that form hydrogen bonds are all conserved highly in the NADP-binding domain and the 12th TM domain which is associated with iron binding. The ligand Drugbank 2154 is etelcalcetide, a calcimimetic drug used for the treatment of hyperparathyroidism.

The mentioned residues of Supplementary Figures S7 in TM12 are associated with the iron-binding domain of STEAP2 proteins in the MSA. The ligand Drugbank138 is lymecycline, a second generation tetracycline antibacterial used for the broad-spectrum treatment of various bacterial infections.

From the images, the protein–ligand complex of ligand 1,543 had the least number of hydrogen bonds between the ligand and protein but had the most number of hydrophobic contacts within the receptor. The summation of these bonds was, therefore, responsible for the observed high-binding energy associated with the complex (Table 4). The ligand Drugbank 1423 is droxidopa, a drug used in treatment of Parkinsonism (Supplementary Figure S8).

**TABLE 4:** Protein–ligand complexes that met the binding energy threshold with respect to the different chains of the protein.

Complex of 01_swissmodel 3D model with different drug compound	Compound name	Homologous chain	Binding energy (kcal/mol)
Drugbank1543	Triptorelin	A	−12.1
Drugbank2	Leuprolide		−11.2
Drugbank2328	1,2 Icosapentoyl-sn-glycero-3-phosphoserine		−8.3
Drugbank2154	Etelcalcetide		−8.2
Drugbank1543	Triptorelin	B	−12.1
Drugbank2	Leuprolide		−11.2
Drugbank2328	1,2 Icosapentoyl-sn-glycero-3-phosphoserine		−8.3
Drugbank138	Lymecycline		−8.1
Drugbank1423	Droxidopa		−7.1
Drugbank1543	Triptorelin	C	−12.1
Drugbank2	Leuprolide		−11.2
Drugbank2328	1,2 Icosapentoyl-sn-glycero-3-phosphoserine		−8.3
Drugbank138	Lymecycline		−8.1
Drugbank1423	Droxidopa		−7.1

Table 4 shows the protein–ligand complexes that met the thresholds of the binding energy.

The complex, 01_SWISS-MODEL and Drugbank 1543 had the least binding energy.

### 3.7 Docking Validation Using Independent Docking Engines

The docking validation by PatchDock produced the same order in terms of scores based on the geometric shape complementarity of solutions. The scores were ranked in the same order as the binding energy measured using AutoDock Vina. The complex of STEAP2 and Drugbank 1543 ligand had the highest score of 11,346 followed by the complex with Drugbank 2 and Drugbank 2328 with scores 11,274 and 11,224, respectively. The least ranked complex was that with Drugbank 3300 (Table5).

**TABLE 5:** PatchDock docking evaluation of docking scores using AutoDock Vina.

STEAP2 complex with	Results’ number	Score	Area	Atomic desolvation energy	Binding energy measured using AutoDock Vina (kcal/mol)	Rank
Drugbank1543	1	11,346	1452.70	−272.92	−12.1	1
	2	11,170	1468.30	−254.68		
	3	1,114	1512.50	−262.71		
Drugbamk2	1	11,274	1460.40	−454.04	−11.2	2
	2	11,088	1490.30	−502.35		
	3	11,086	1469.60	−501		
Drigbank2328	1	11,224	1375.80	-421.80	−8.3	3
	2	10,568	1383.10	−501.26		
	3	10,506	1321.70	−462.1		
Drugbank2154	1	9,240	1222.30	−769.57	−8.2	4
	2	9,096	1,187.60	−734730		
	3	9,094	1132.30	−453.96		
Drurgbank138	1	7,074	814.80	−421.80	−8.1	5
	2	7,016	797.00	−382.80		
	3	6,970	841.00	−373.47		
Drugbank1423	1	3,300	358.90	−133.28	−7.1	6
	2	3,294	357.70	−125.81		
	3	3,294	363.30	−122.28		

Table 5 shows the top 3 solution of each complex ranked in order of their scores by PatchDock. The area stands for the approximate interface area of the complex and atomic desolvation energy is measured according to Zhang et al. (2001)

The exact docking poses from AutoDock Vina could not be reproduced by PatchDock despite maintaining the same ranking order. Another docking engine, AutoDock4, could not reproduce the exact docking poses.

The results from the ProBiS analysis of STEAP2 for potential binding sites found STEAP4 as the most locally and structurally similar protein as it was also the used template for homology modeling. The most structurally conserved residues in binding site one, for small molecules, appeared red and were in the NADP-binding domain. All six identified binding ligands were NADP’s with confidences of 4.21–3.58. Binding site two, for proteins, had all amino acids unconserved as they appeared blue and confidence from 2.42 to 2.29. No binding sites were found for nucleic acids and ions (Supplementary Figure S9A).

Consistent with these findings were those from PrankWeb whose pocket 1 was HEM binding (pocket score 24.48), 2 (pocket score 10.48) was NADP binding with the most conserved residues, pockets 3 and 6 with pocket scores 6.26 and 3.09, respectively, were also within the NADP-binding domain. Pockets 4 and 5 were all within the FAD-binding domain with scores 5.86 and 3.75, respectively. Surface pockets 4 and 5 and pocket 3 were shown to be in close proximity with docked ligands, triptorelin and leuprolide (Supplementary Figure S10).

The results from CAVIAR analysis for binding sites found 7 cavities, cavity 5 with ligandability score 0.8 lays in the most conserved residues of the chain that also bind NADP, 1 and 6 lay in the HEM-binding domain that is not very conserved with ligandability scores of 0.6 and 0.8, respectively, and 2 with ligandability score 0.8 lays in the FAD-binding domain. Cavity 2/pocket 2 was close to bound ligands triptorelin and leuprolide. Cavities with ligandability score of 0.8–1 are considered easy to the ligand and of these, cavity 5 had the best score, size (109 A), and degree of conserved residues in comparison with conservation residue score results from PrankWeb. These results are detailed in a text file of cavity identification and Supplementary Figure S9.

## 4 Discussion

Our study aimed to identify potential compounds that could be used in the development of anti-prostate cancer drugs that target STEAP2 using molecular docking. To our knowledge at the time, there are no studies in the literature that have used this approach with the aim of treating prostate cancer. Findings from our study showed that drugs such as triptorelin and leuprolide earlier prescribed for advanced prostate cancer could be effective in early-stage diseases with a more defined mode of action.

With a modeled homolog and docking process of a pool of approved candidates from the drug bank, a number of drug compounds that formed significant interactions with the homology model gave insight to some of the current drugs that could be repurposed for the treatment of prostate cancer.

The best homology model of STEAP2 was generated by the SWISS-MODEL as shown by the results from assessment by the 5 evaluation tools used (Figure 8A). SWISS-MODEL, in a study by Wallner, has been shown to accurately model homologs whose templates are of as low as 40% similarity (Wallner and Elofsson, 2005) suggesting that the modeling engine is reliable.

On model analysis, a lower score below 100% in the favored region on a Ramachandran plot shows that there are residues whose phi and psi angles are not sterically possible when modeled (Ramachandran et al., 1963). A lower percentage of residues in the favored region, therefore, indicates a poor homology model as shown for models from Robetta and I-Tasser. Our best homology model, by SWISSMODEL, had 96.7% of the protein residues being under the favored region and this shows that most of the protein residues are properly sterically placed in 3D space.

From the docking results, ligand Drugbank 1543 had the least binding energy and highest affinity from a selected set of 2,466 ligands. Having the least binding energy means the complex is more naturally stable due to affinity between the two constituent molecules with no or less external energy applied. The ligand formed its hydrogen bond with a residue Ser372 in the transmembrane domain of the receptor molecule as most of its other non-hydrogen bond interactions. From a key of the Drugbank, Drugbank 1543, accession number DB06825, generic name triptorelin is a synthetic decapeptide gonadotrophin releasing hormone (GnRH) agonist. The drug is proven for the palliative treatment of advanced stage prostate cancer (N A et al., 2015). With the results from this study, this drug could potently be used to treat early-stage diseases targeting STEAP2 that is shown to be responsible for disease progression as shown in the study by Whiteland et al. (2014). This still requires corroboration from further studies in the future.

The study by Ravenna et al. (2000) demonstrates that triptorelin has an inhibitory-stimulatory effect on lymph node carcinoma of the prostate (LNCaP) cells at lower and high doses, respectively. In the experiment, in Pc3 cell lines, only low affinity but high capacity receptors were expressed as a biological reaction to the drug. Both low and moderately high affinity with high and low capacity receptors, respectively, were expressed in LNCaP cells. This shows the potency of triptorelin and its binding energy in the treatment of prostate cancer at different stages. Since STEAP2 is present in both cell types and given the binding relationship between the protein and drug, this study suggests a mode of action of the latter.

Drugbank 2 accession number DB00007 generic name leuprolide is a synthetic 9 residue peptide analog of GnRH shown to have a quicker onset of action in achieving serum testosterone levels equivalent to medically castrated men compared to triptorelin (Heyns et al., 2003). The findings from this study suggest STEAP2 as a target route for action.

Data from the protein atlas places brain tissue (pituitary gland, cerebral cortex, and basal ganglia) third with the STEAP2 content after the ovary and vagina [(Korkmaz et al., 2005), (Porkka et al., 2002b)] In men, brain tissue becomes second after the prostate though with a significant difference in tissue STEAP2 expression. This could account for some of the potency of the GnRH receptor analog agonists in treating prostate cancer as mentioned earlier.

Drugbank 2328, accession number DB14096, 1,2 icosapentoyl-sn-glycero-3-phosphoserineis an approved experimental drug for the treatment of Corona Virus Disease-19 (COVID-19) (Kouznetsova et al., 2020). Drugbank 138, accession number, DB00256, lymecycline is a broad spectrum second generation tetracycline antibacterial used for the treatment of susceptible bacterial infections such as acnes (Zouboulis and Piquero-Martin, 2003). Drugbank 1423, accession number DB06262, generic name, droxidopa, a precursor for noradrenaline and is used in the treatment of Parkinsonism (Longardner et al., 2020). Drugbank 2154, accession number DB12865, generic name etelcalcetide is a calcimimetic drug used for the treatment of secondary hyperparathyroidism in patients having hemodialysis. From the findings of this study, these drugs could treat prostate cancer by binding to and inhibiting the catalytic role of STEAP2 that is responsible for disease progression as seen by the result in this study. Further investigation on their role in the treatment of prostate cancer is still warranted.

We presume the exact docking poses could not be reproduced by AutoDock4 because the engine requires the use of a single chain of the homo-3-mer for the protein ligand docking vis-à-vis the 3 identical chains used in the docking by AutoDock Vina. The coherence in ranking order of the protein–ligand complex stability between AutoDock Vina and PatchDock, however, suggests the complexes are rightfully ordered.

The metalloreductase activity of STEAP2 occurs across its transmembrane domains but these are neither binding sites nor druggable regions of the protein from binding site analysis using ProBiS, PrankWeb, and CAVIAR (Konc and Janežič, 2010b; Jendele et al., 2019; Marchand et al., 2021; Rocha et al., 2021). These results demonstrate that the cytoplasmic NADP-binding domain is a potential binding site but it being intracellular reduces its druggability. The residues Ser-3712 and Gly-369 do not fall in any of the mentioned binding pockets but are in close proximity with the FAD-binding pocket. A bound ligand in this position as is leuprolide and triptorelin could give steric hindrance to the FAD and consequently the overall function of the metalloreductase.

The production of the STEAP2 should, therefore, be both suppressed in the prostate and where possible, a potent ligand should bind to it to block its role in prostate cancer progression.

Its role in metal Fe3+ and Cu2+ reduction for transmembrane transfer into cells can be carried on by STEAP3 metalloreductase associated with prostate cancer tumor suppression through induced apoptosis (Steiner et al., 2000; Zhang et al., 2001; Rocha et al., 2021). Other STEAP metalloreductases such as STEAP4 and STEAP1 linked with prostate cancer progression as shown in studies by Gomes et al. (2014) and Jin et al. (2015) can be targets for suppression and or inhibition for their role in prostate cancer progression using inhibitory ligands.

The findings from this study give conjecture on the mechanistic role of triptorelin and leuprolide among others if STEAP2 was a target in the treatment of prostate cancer right from the early-stage disease. Targeting STEAP2 may, therefore, lead to the development of new anti-prostate cancer therapies in the future with further studies.

Due to time and cost limitations we were unable to successfully validate the docking poses but the binding energy rankings of the top ligands using different and independent docking engines.

We recommend that future work should include reproducing the docking poses using other commercial and/or free docking engines in addition to molecular dynamics simulations. The docking engine used (AutoDock Vina) is, however, documented to be one of the best at the time as shown in the study by Sousa et al. (2013). Additionally, we also recommend further analysis in terms of *in vitro* assays to validate the results from this *in silico* study on growth and viability changes in the presence of these drugs and whether there are genes, gene products, and or enzymes that could potentially upregulate STEAP2 in its signaling pathway leading to its role in the development and progression of prostate cancer.

## Data Availability

The original contributions presented in the study are included in the article/Supplementary Material, further inquiries can be directed to the corresponding author.
