# Supplementary material for: A Molecular Docking Study of Human STEAP2 for the Discovery of New Potential Anti-Prostate Cancer Chemotherapeutic Candidates
Source: Front Bioinform. 2022 May 24;2:869375. doi: 10.3389/fbinf.2022.869375 (PMC9580961; doi:10.3389/fbinf.2022.869375)
Supplement: Supplementary file 2 [file Table1.DOCX]

Supplementary Material

## Supplementary Figures


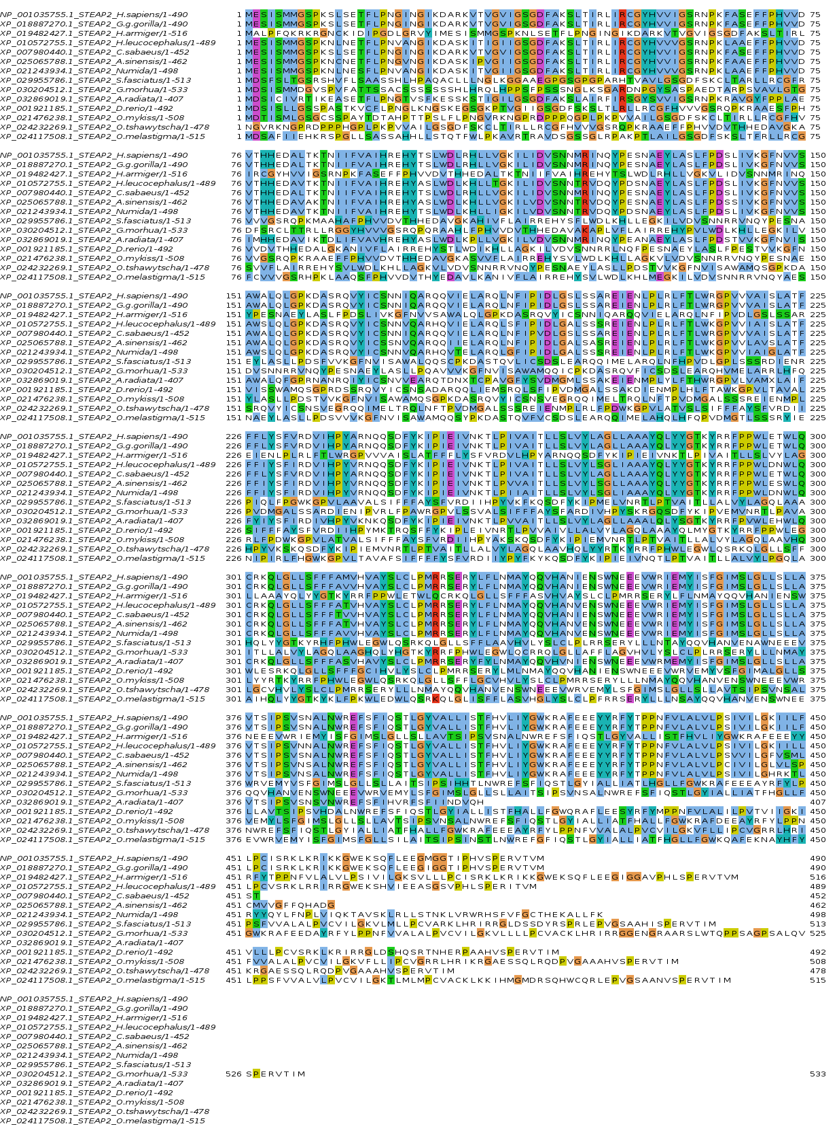


**Supplementary Figure 1.**

**Supplementary Figure 2.** A bar graph showing the differential tissue expression of STEAP2 RNA tissue data reported as scaled tags per million by FANTOM5 dataset (source: <https://www.proteinatlas.org/ENG00000157214-STEAP2>). The graph shows that STEAP2 mRNA is most abundantly expressed in prostate tissue followed by ovarian and vaginal tissue.

**Supplementary Figure 3.** A bar graph showing the differential tissue expression of STEAP2 in mean protein coding transcripts per million by HPA dataset (source: <https://www.proteinatlas.org/ENG00000157214-STEAP2)>. The prostate tissue had a significantly high STEAP2 in mean protein coding transcripts per million as compared to all other listed body tissues followed by the appendix and parathyroid gland.

**Supplementary Figure 4.** A bar graph showing the differential tissue expression of STEAP2 by RNA sequence tissue reported in mean protein coding transcripts per million shown by GTex dataset (source: <https://www.proteinatlas.org/ENSG00000157214-STEAP2/tissue>). Prostate tissue has a highly significant content of STEAP2 RNA compared to all other tissues followed by lung and pituitary glands.


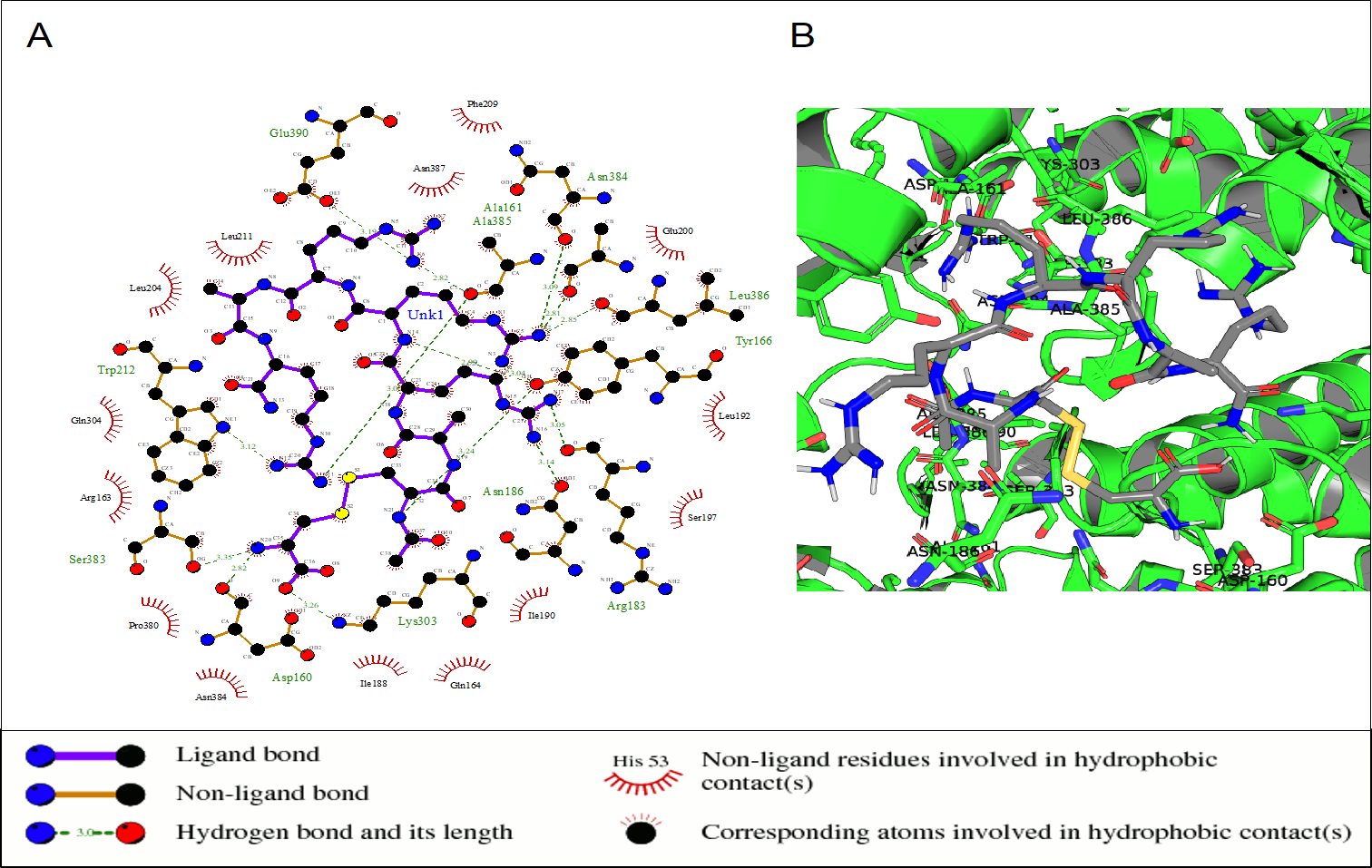


**Supplementary Figure 5**. In the above complex, receptor residues Glu-390, Ala-161,386, Asn-186,384, Lue-386, Tyr-166, Trp-212, Ser-383, Lys-303, Arg-183 and Asp160 all form hydrgen bonds with ligand DrugBank2154. The residues are majorly from the NADP binding dominan and the 12^th^ TM domain


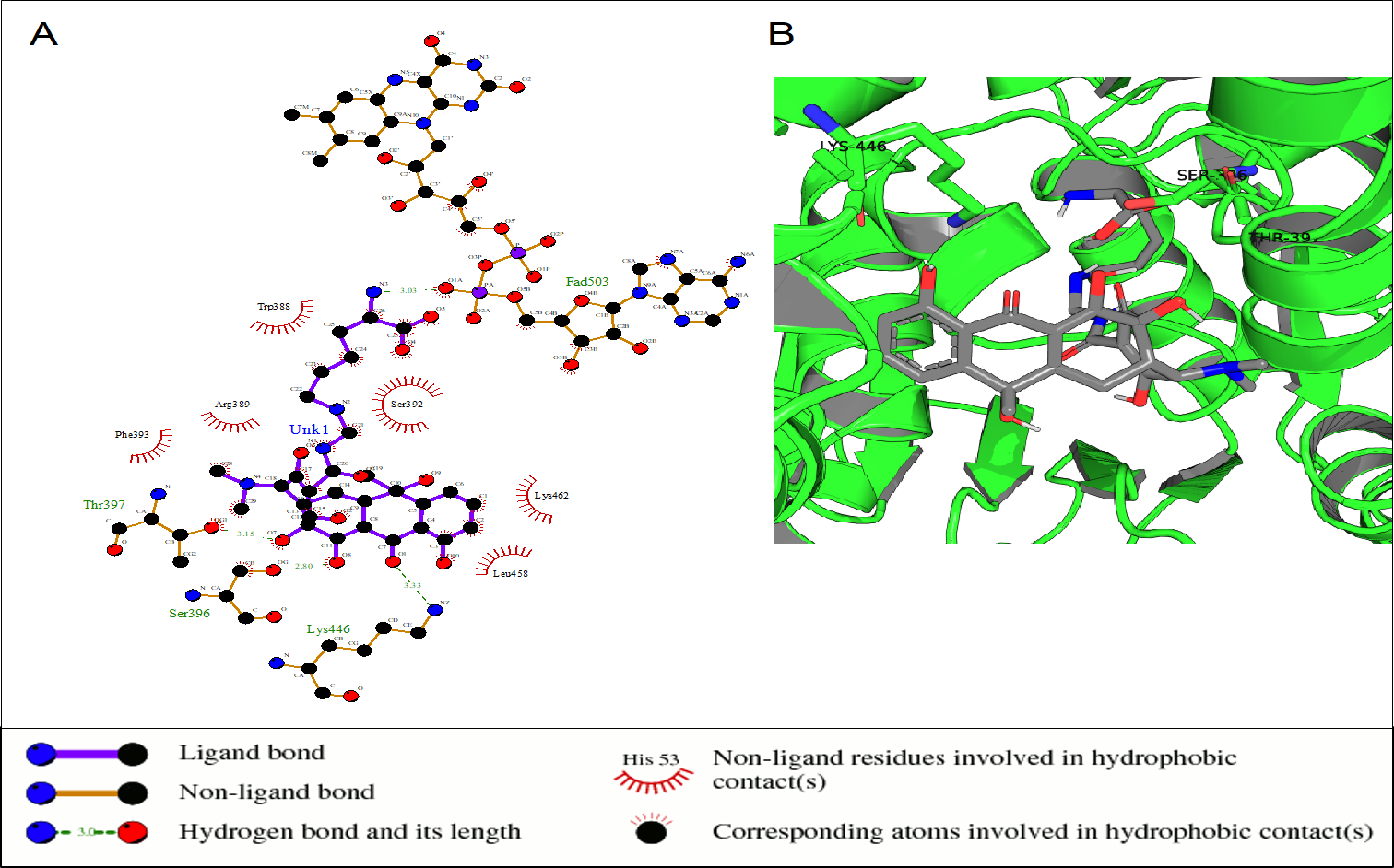


**Supplementary Figure 6**. In the above complex, receptor residues Thr-397, Ser-396 (TM12) and Lys-446 (TM17) form hydrogen bonds with the ligand Drugbank138


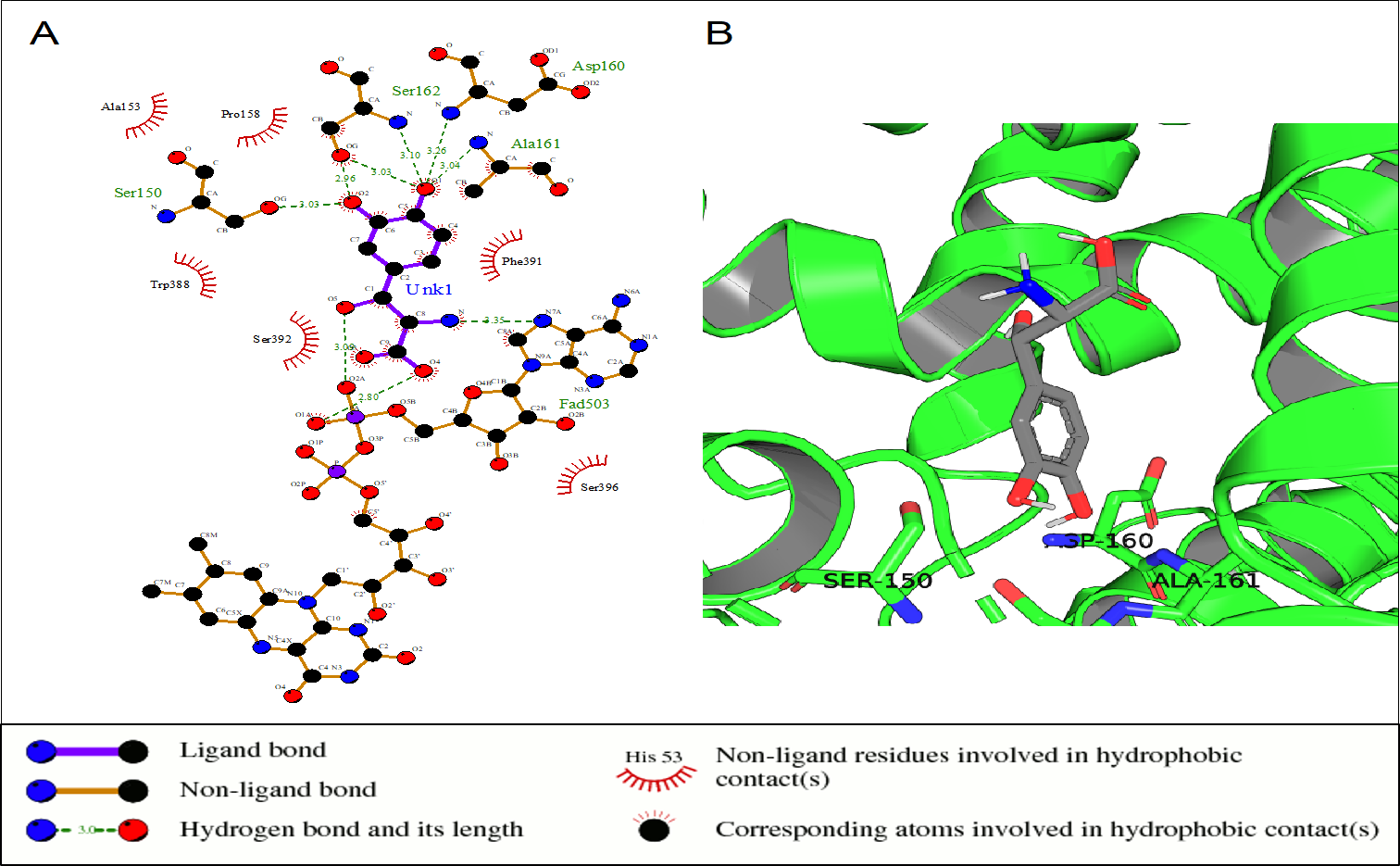


**Supplementary Figure 7**. In the above complex (A), residues Asp-160, Ser-162,150 and Ala-161 all within the NADP binding domain form hydrogen bonds with the ligand DrugBank1423*.*
