# Supplementary material for: A Molecular Docking Study of Human STEAP2 for the Discovery of New Potential Anti-Prostate Cancer Chemotherapeutic Candidates
Source: Front Bioinform. 2022 May 24;2:869375. doi: 10.3389/fbinf.2022.869375 (PMC9580961; doi:10.3389/fbinf.2022.869375)
Supplement: Supplementary file 3 [file Table3.DOCX]

Supplementary Material

## Supplementary Figures


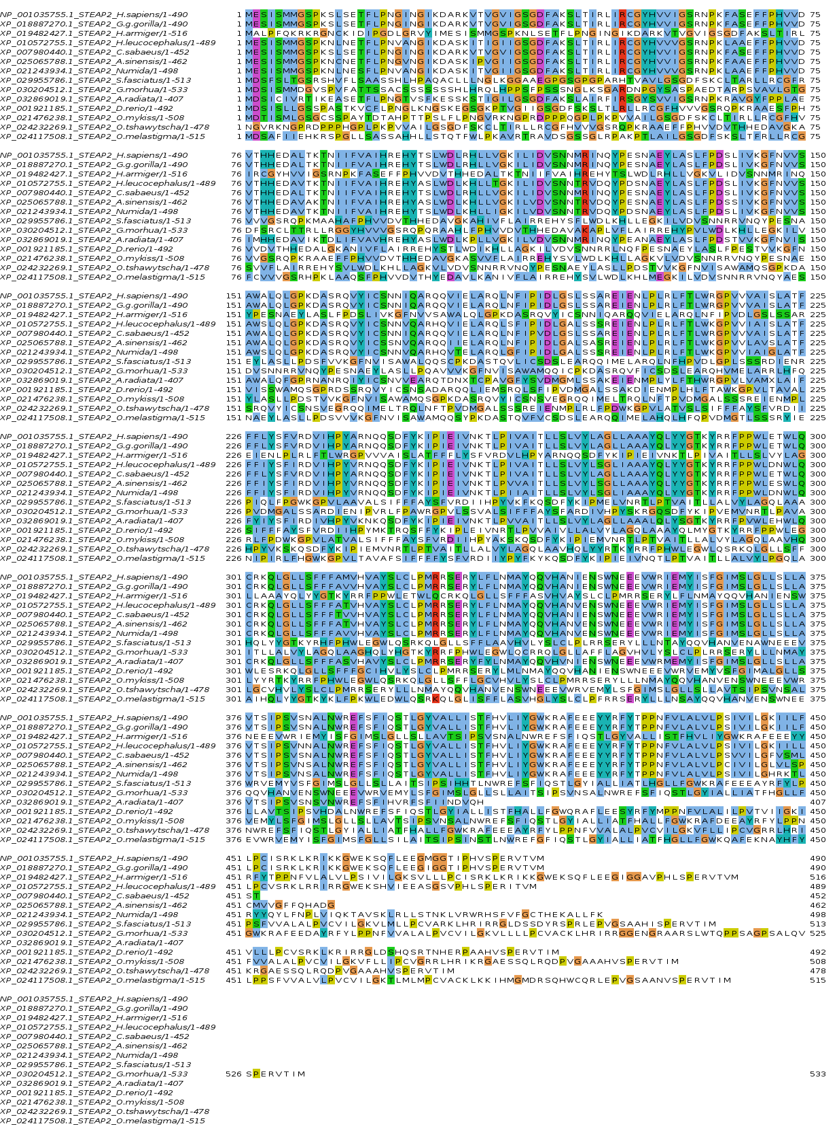


**Supplementary Figure 1.**

**Supplementary Figure 2.** A bar graph showing the differential tissue expression of STEAP2 RNA tissue data reported as scaled tags per million by FANTOM5 dataset (source: <https://www.proteinatlas.org/ENG00000157214-STEAP2>). The graph shows that STEAP2 mRNA is most abundantly expressed in prostate tissue followed by ovarian and vaginal tissue.

**Supplementary Figure 3.** A bar graph showing the differential tissue expression of STEAP2 in mean protein coding transcripts per million by HPA dataset (source: <https://www.proteinatlas.org/ENG00000157214-STEAP2)>. The prostate tissue had a significantly high STEAP2 in mean protein coding transcripts per million as compared to all other listed body tissues followed by the appendix and parathyroid gland.

**Supplementary Figure 4.** A bar graph showing the differential tissue expression of STEAP2 by RNA sequence tissue reported in mean protein coding transcripts per million shown by GTex dataset (source: <https://www.proteinatlas.org/ENSG00000157214-STEAP2/tissue>). Prostate tissue has a highly significant content of STEAP2 RNA compared to all other tissues followed by lung and pituitary glands.


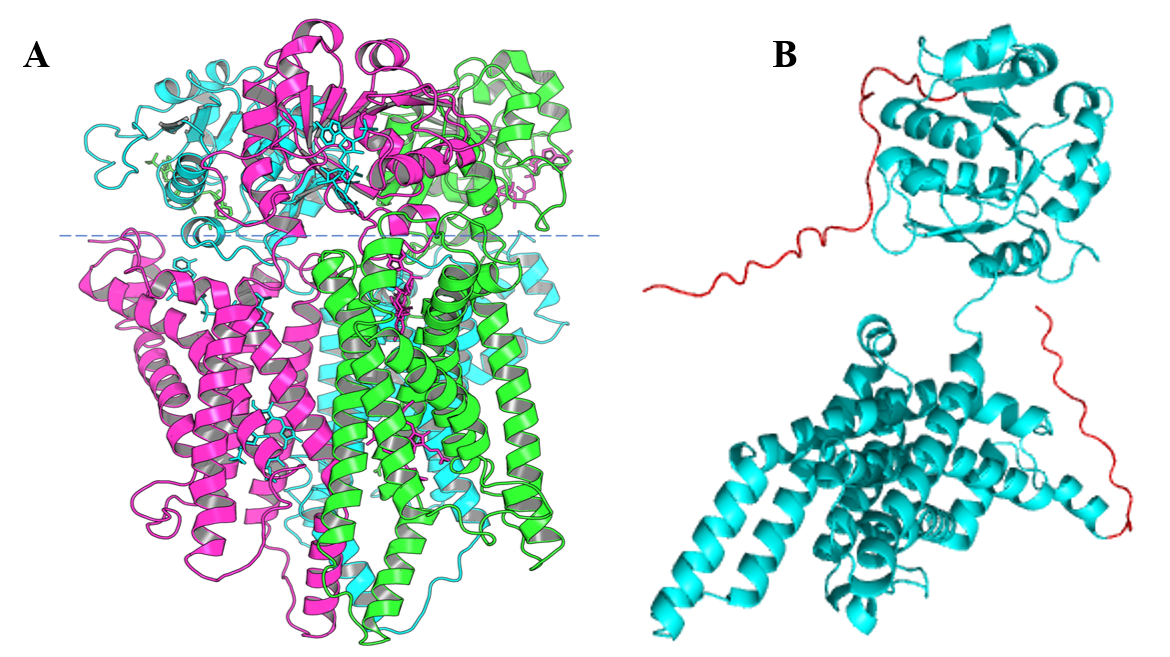


**Supplementary figure 5**: Comparison of structure prediction of STEAP2 homology model (**A**) and ab initio folded structure (**B).** The homology model has three chains (colored purple, cyan and green) as its template structure STEAP4. Other STEAP metalloreductases have 9 chains (STEAP1) and 2 chains(STEAP3). The ab initio folded structure is a single chain (colored cyan) and has poorely modelled residues (1 to 32 and 474 to 490)in loops colored red.

The STEAP2 490 amino acid model from AlphaFold2 scored best on the ProZA, QMEAN analysis with -6.65 and -2.54 Z scores respectively, and a 96.818 percentage of amino acids in the favoured region on a Ramachandran plot. It, however, was a monomer and had regions of long loops with very low predicted local distance difference test scores up to 28.05 (on a scale of 100). In comparison to a similar structurally solved STEAP4 (PDB Id; 6hcy) it had a RMSD of 0.857 quite different from 0.086 of the homology modelled homo-3-mer structure of choice of the study. These results suggest the homology model from SWISSMODEL was a better predicted structure than the ab initio folded one from AlphaFold2.

| **MODELLING ENGINE** | **STRUCTURE**  **STOICHIOMETRY** | **ProSA**  **(Z-Score)** | **QMEAN**  **(Z- Score)** | **RAMPAGE**  **FAVOURED ALLOWED OUTLIER** | | | **AA MODELLED** | **RMSD WITH 6HCY** |
| --- | --- | --- | --- | --- | --- | --- | --- | --- |
| ALPHAFOLD | MONOMER | -6.65 | -2.54 | 96.8 | 1.8 | 1.4 | 490 | 0.857 |
| **SWISSMODEL** | **HOMO-3-MER** | **-6.33** | **-3.06** | **94.4** | **4.8** | **0.2** | **437** | **0.086** |


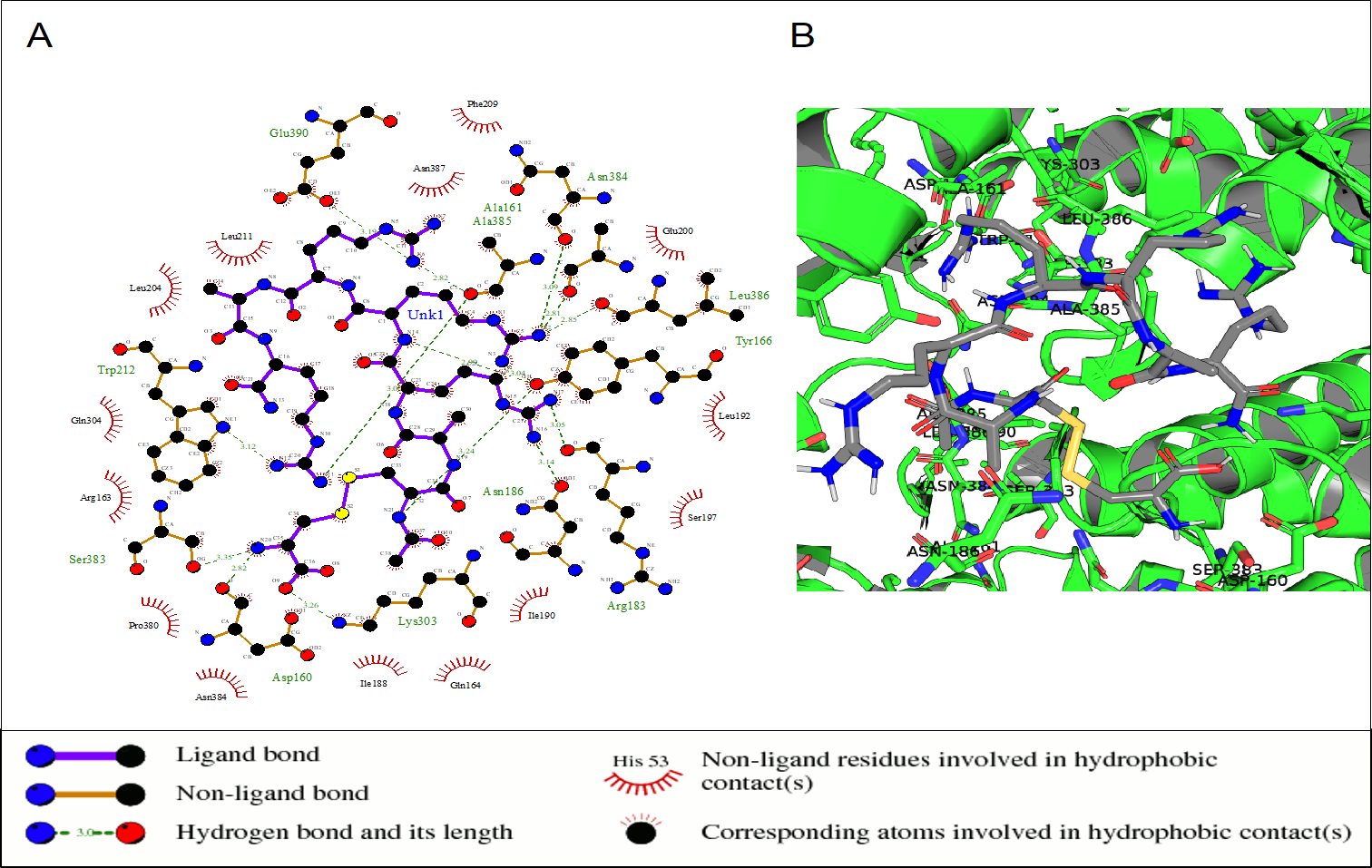


**Supplementary Figure 6**. In the above complex, receptor residues Glu-390, Ala-161,386, Asn-186,384, Lue-386, Tyr-166, Trp-212, Ser-383, Lys-303, Arg-183 and Asp160 all form hydrgen bonds with ligand DrugBank2154. The residues are majorly from the NADP binding dominan and the 12^th^ TM domain


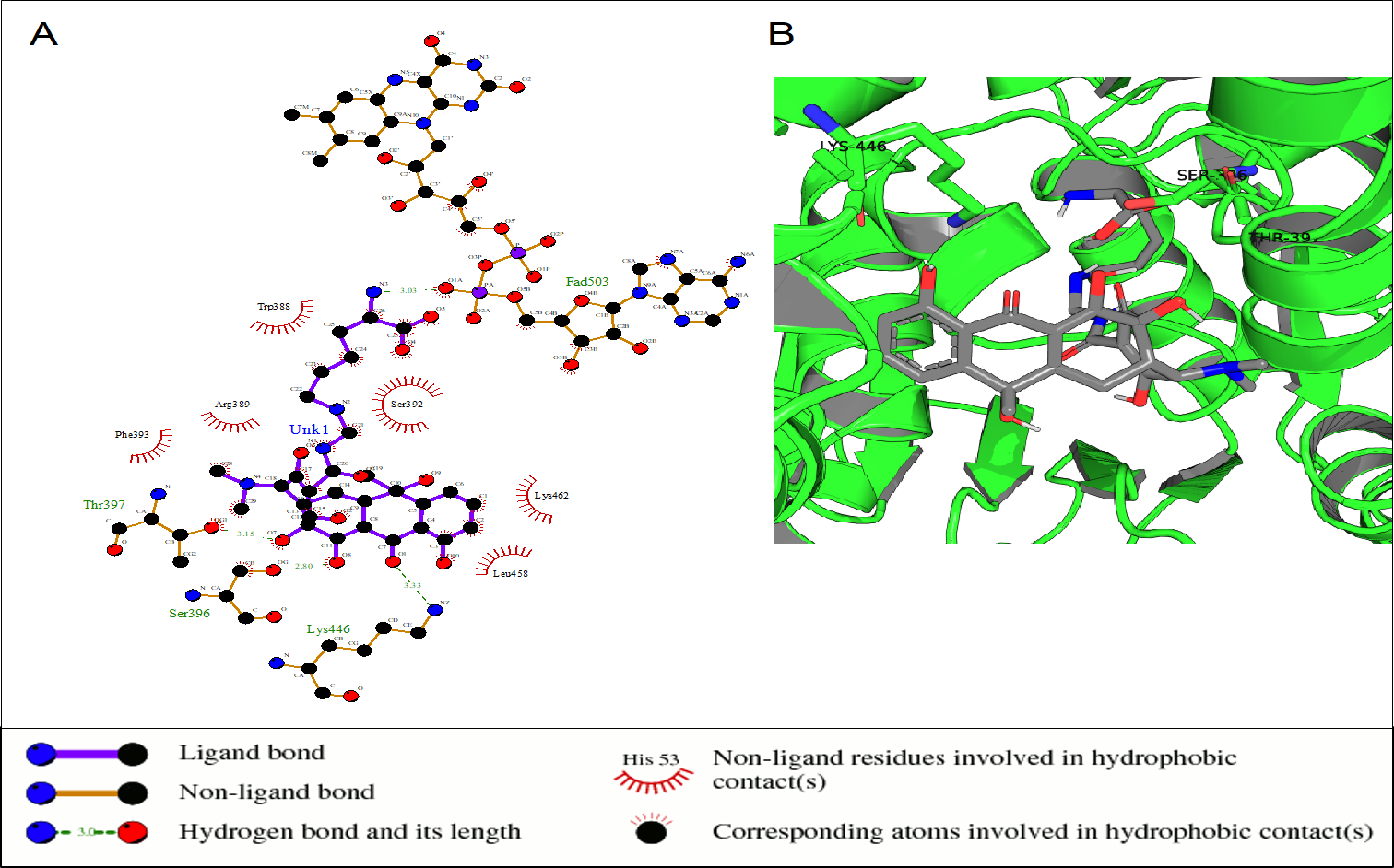


**Supplementary Figure 7**. In the above complex, receptor residues Thr-397, Ser-396 (TM12) and Lys-446 (TM17) form hydrogen bonds with the ligand Drugbank138


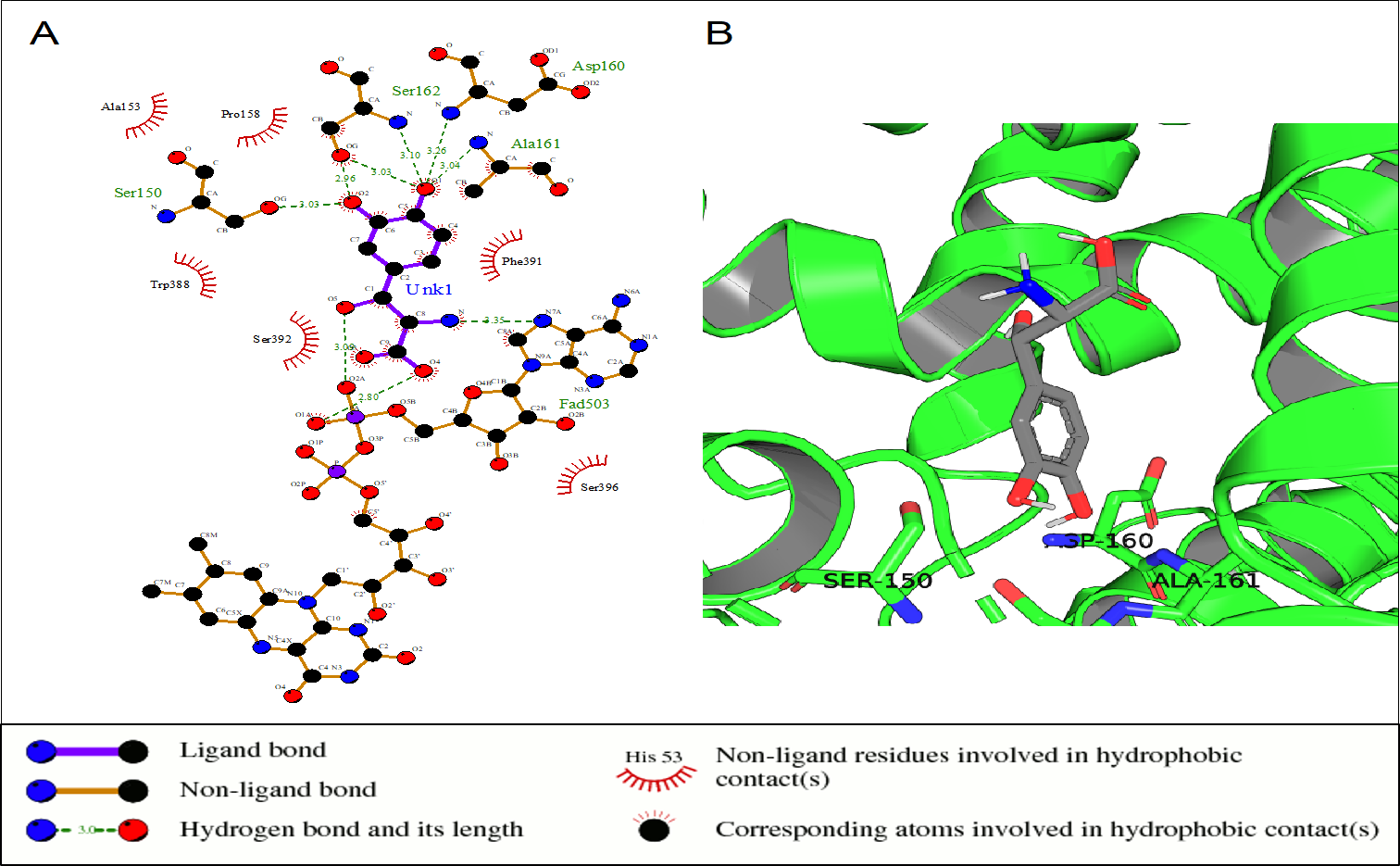


**Supplementary Figure 8**. In the above complex (A), residues Asp-160, Ser-162,150 and Ala-161 all within the NADP binding domain form hydrogen bonds with the ligand DrugBank142


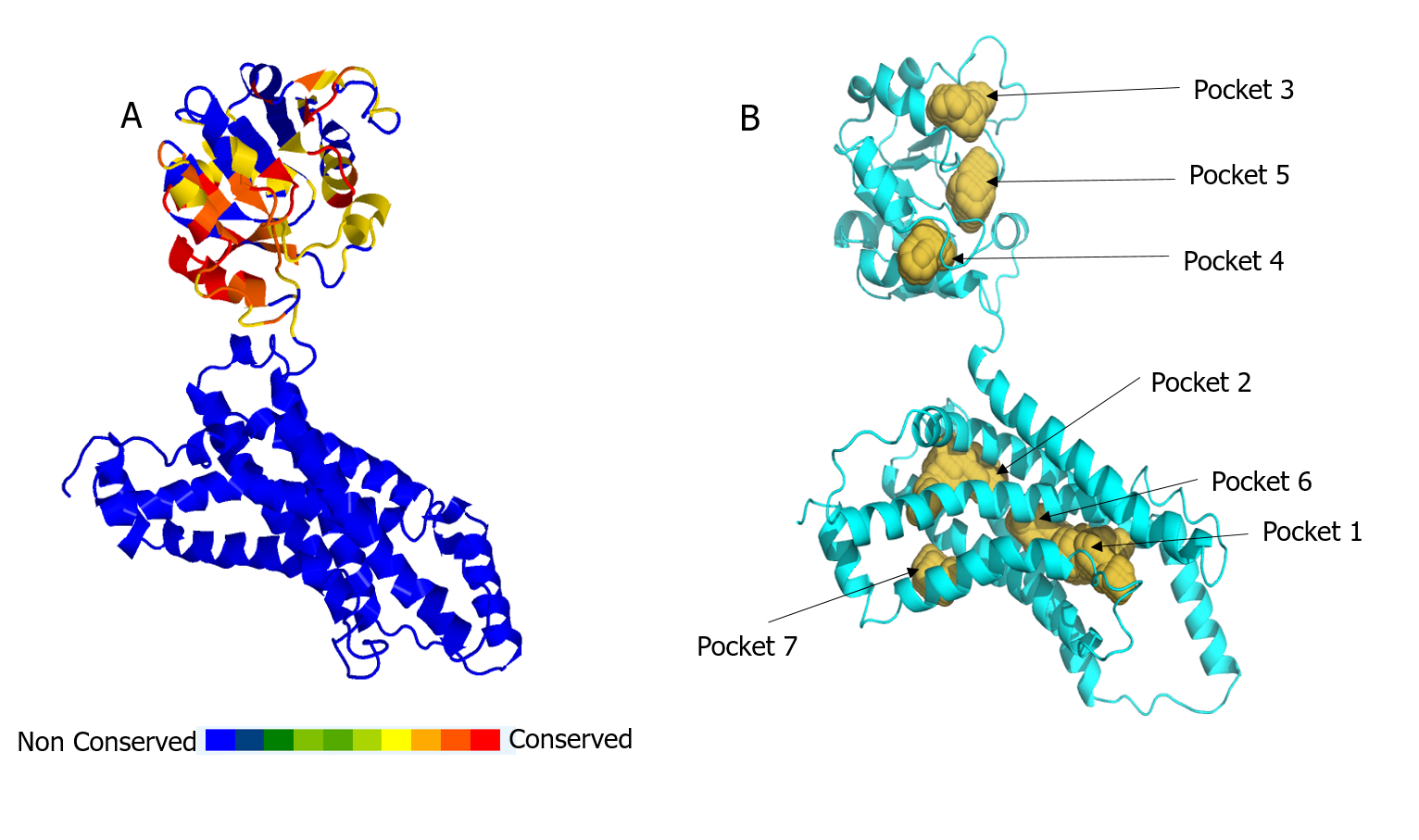


**Supplementary Figure 9.** Analysis of STEAP2 Chain A for potential binding sites using ProBiS (A) and CAVIAR (B). Ligand binding pockets 3,4 and 5 lie within very structurally conserved residues while pockets 1,2 6 and & lie within non conserved residues.


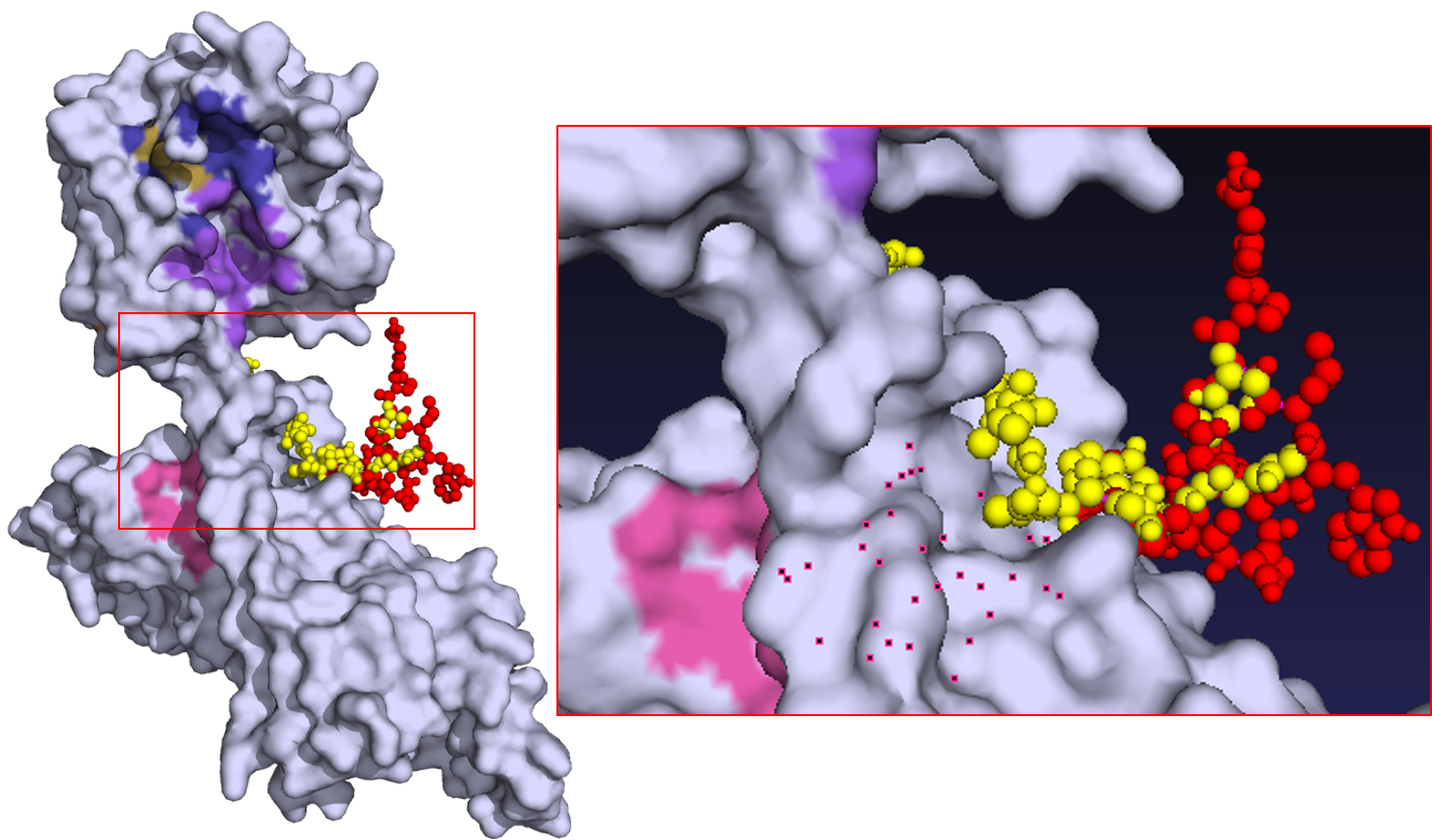
**Supplemetary Figure 10**: Comparison of docking results with pocket identification from PrankWeb. Triptorelin (ligand) is shown in red spheres and Leuprolide shown in yellow spheres are in close proximity with surface pockets 5 (shown with red dots) and 4 (in pink) and pocket 3 ( in purple). Surface pockets 4 and 5 are closely linked to pocket 2 identified from CAVIAR.
